# Multiple routes to fungicide resistance: Interaction of *Cyp51* gene sequences, copy number and expression

**DOI:** 10.1111/mpp.13498

**Published:** 2024-09-20

**Authors:** Corinne J. Arnold, Emily A. (Meyers) Hahn, Rebecca Whetten, Laetitia Chartrain, Jitender Cheema, James K. M. Brown, Christina Cowger

**Affiliations:** ^1^ John Innes Centre, Norwich Research Park Norwich UK; ^2^ Department of Entomology and Plant Pathology North Carolina State University Raleigh North Carolina USA; ^3^ United States Department of Agriculture‐Agricultural Research Service, Department of Entomology and Plant Pathology North Carolina State University Raleigh North Carolina USA; ^4^ Present address: Camena Bioscience, Chesterford Research Park Cambridge UK; ^5^ Present address: Wisconsin Crop Innovation Center University of Wisconsin 8520 University Green Middleton Wisconsin USA

**Keywords:** *Blumeria graminis* f. sp. *tritici*, *Cyp51*, demethylation inhibitors, *Erg11*, fungicide resistance, heteroallelism, powdery mildew, triazoles

## Abstract

We examined the molecular basis of triazole resistance in *Blumeria graminis* f. sp. *tritici* (wheat mildew, Bgt), a model organism among powdery mildews. Four genetic models for responses to triazole fungicides were identified among US and UK isolates, involving multiple genetic mechanisms. Firstly, only two amino acid substitutions in CYP51B lanosterol demethylase, the target of triazoles, were associated with resistance, Y136F and S509T (homologous to Y137F and S524T in the reference fungus *Zymoseptoria tritici*). As sequence variation did not explain the wide range of resistance, we also investigated *Cyp51B* copy number and expression, the latter using both reverse transcription‐quantitative PCR and RNA‐seq. The second model for resistance involved higher copy number and expression in isolates with a resistance allele; thirdly, however, moderate resistance was associated with higher copy number of wild‐type *Cyp51B* in some US isolates. A fourth mechanism was heteroallelism with multiple alleles of *Cyp51B*. UK isolates, with significantly higher mean resistance than their US counterparts, had higher mean copy number, a high frequency of the S509T substitution, which was absent from the United States, and in the most resistant isolates, heteroallelism involving both sensitivity residues Y136+S509 and resistance residues F136+T509. Some US isolates were heteroallelic for Y136+S509 and F136+S509, but this was not associated with higher resistance. The obligate biotrophy of Bgt may constrain the tertiary structure and thus the sequence of CYP51B, so other variation that increases resistance may have a selective advantage. We describe a process by which heteroallelism may be adaptive when Bgt is intermittently exposed to triazoles.

## Graphical abstract

Azole resistance in wheat mildew increases by mutations in *CYP51B*, higher gene expression and copy number, and heteroallelism: both resistance and sensitivity alleles being present in one isolate.

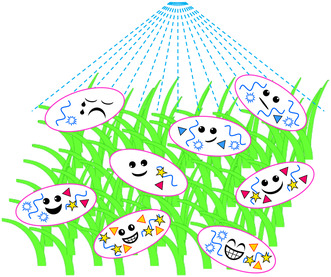

## 1 INTRODUCTION

Sterol demethylation inhibitors (DMIs), especially triazoles, have been used worldwide since the 1970s to control fungal diseases caused by ascomycete and basidiomycete pathogens in medicine and agriculture (Oliver, 2014), including powdery mildew of many crops (Blatter et al., 1998; Sheehan et al., 1999; Ziogas & Malandrakis, 2015). Triazoles form the largest class of fungicide used to control fungal pathogens of crops, farm animals and humans worldwide. DMIs inhibit the CYP51 lanosterol 14α‐demethylase, also known as ERG11, a cytochrome P450 enzyme in the ergosterol biosynthesis pathway (Hull et al., 2012) encoded by nuclear *Cyp51* genes (Ma & Michailides, 2005). Powdery mildew fungi, including *Blumeria graminis*, a pathogen of cereals and grasses, have the *Cyp51B* member of this gene family. The principal membrane sterol of powdery mildews is the unusual compound ergosta‐5,24(24^1^)‐dien‐3β‐ol (PubChem compound identifier 314582; Loeffler et al., 1992), differing from the much more common ergosterol (PubChem CID 444679) in lacking one of the two double‐bonds in the sterol B ring and in the position of a double‐bond in the side chain. Nevertheless, ERG11/CYP51 is an essential enzyme in powdery mildews as in other ascomycetes.

Resistance or reduced sensitivity to DMI fungicides is a quantitative trait that has evolved gradually in many fungi, including *B. graminis* (Blatter et al., 1998; Tucker et al., 2019; Wyand & Brown, 2005). There are three known mechanisms of resistance, also known as insensitivity, involving the *Cyp51* gene: sequence mutations, increased gene copy number and increased gene expression (Ziogas & Malandrakis, 2015). Increased expression of transporter genes is another mechanism of DMI resistance in several fungi, not associated with variation in *Cyp51*. DMI‐resistant fungi may have combinations of two or more resistance mechanisms, leading to complete or partial cross‐resistance between different DMIs in various pathogens, including powdery mildews (Blatter et al., 1998; Cools et al., 2011; Marichal et al., 1997; Rallos & Baudoin, 2016; Sanglard et al., 1995; Stammler et al., 2009; Venkateswarlu et al., 1995; Wyand & Brown, 2005). The combination of *Cyp51* mutation with overexpression can cause high resistance and strong cross‐resistance to DMI fungicides, including triazoles (Cools et al., 2013).

Many amino acid changes within the CYP51 protein have been identified in fungi showing a range of levels of resistance to DMIs (Cools et al., 2011; Rallos & Baudoin, 2016; Sanglard et al., 1995). Many of them alter the structure and shape of the binding pocket such that the enzyme is still functional but DMI molecules are unable to bind normally (Becher & Wirsel, 2012; Chartrain & Brown, 2023; López‐Ruiz et al., 2011; Tucker et al., 2019). The most common substitution is Y137F (Becher & Wirsel, 2012), [Y137F] in the unified nomenclature for fungicide resistance mutations (Mair et al., 2016); i.e. [Y137F], homologous to Y136F in *B. graminis*. This is predicted to increase the hydrophobicity of the DMI binding site (Délye et al., 1997). *Zymoseptoria tritici* isolates resistant to triazoles such as epoxiconazole (Cools et al., 2011) have a wide range of mutations in *Cyp51*, often occurring in various combinations rather than individually. Many *Z. tritici* isolates containing F137, homologous to F136 in *B. graminis*, also had S524T (homologous to amino acid 509 in *B. graminis*), which compensated for the fitness penalty of the Y137F substitution (Cools & Fraaije, 2008; Cools et al., 2010, 2011). Modelling of the CYP51 protein with changes identified in *Z. tritici* isolates showed a loss of two β‐sheets that contributed to the structure of the protein's active site, causing it to have a more open conformation (Cools et al., 2011). This resulted in DMI fungicide molecules binding with lower affinity.

By contrast, few *Cyp51* mutations have been reported in powdery mildews. In *B. graminis* and *Erysiphe necator* (grapevine powdery mildew), all DMI‐sensitive isolates tested had Y136 while those resistant to a triazole fungicide, triadimenol, often but not always contained Y136F (hereafter F136) (Délye et al., 1997, 1998; Frenkel et al., 2015; Wyand & Brown, 2005). Among isolates with Y136F, levels of resistance were highly variable, implying the existence of additional resistance mechanisms. The Y136F substitution has also been found in *B. graminis* f. sp. *hordei* (Bgh, barley powdery mildew) in Western Australia, and the S509T substitution was found in combination with Y136F in extremely DMI‐resistant isolates (Tucker et al., 2019). A K147Q substitution [K148Q] was found in highly triazole‐resistant Bgh isolates (Wyand & Brown, 2005).

A second mechanism for DMI resistance is overexpression of the *Cyp51* gene. With an excess of functional CYP51 protein, DMI molecules may be too scarce to effectively inhibit sterol production. This mechanism has arisen in a plethora of fungi due to alterations of the promoter region, for example tandem repeats of an activator site (Hamamoto et al., 2000) or insertions (Garcia‐Effron et al., 2008; Hamamoto et al., 2000; Ma et al., 2006; Schnabel & Jones, 2001; Villani et al., 2016). In *Cercospora beticola*, overexpression of *Cyp51* caused resistance despite an absence of amino acid substitutions in the CYP51 protein sequence (Nikou et al., 2009).

In some fungi, *Cyp51* overexpression has evolved by an increase in the gene copy number (Jones et al., 2014; Marichal et al., 1997), including the plant‐pathogenic genera *Fusarium*, *Aspergillus* and *Rhynchosporium* (Cools et al., 2013; Hawkins et al., 2014; Song et al., 2018). More prolific expansion of the gene family was present in *E. necator*, where two to 14 copies of the *Cyp51* gene containing F136 were found in isolates from fungicide‐treated vineyards, while isolates from untreated vineyards only had one copy of the gene containing wild‐type Y136. Increased copy number was associated with increased *Cyp51* gene expression (Jones et al., 2014). The highest levels of DMI resistance were present in isolates with a mixed genotype, where some copies of the *Cyp51* gene had Y136 and others F136 (Rallos & Baudoin, 2016).

*Blumeria graminis*, an obligate ascomycete, undergoes approximately annual sexual recombination and proliferates during growing seasons by polycyclic production of easily windblown conidia. These traits give the fungus an enormous effective population size and a high capacity to adapt to host varieties' resistance or fungicides (Brown, 1994; Cowger et al., 2016). DMIs are among the few classes of fungicide effective in controlling cereal powdery mildew and have been applied intensively to European cereal crops since the late 1970s (Limpert, 1987). As a result, widespread DMI resistance developed in European populations of Bgh and *B. graminis* f. sp. *tritici* (Bgt, wheat powdery mildew) (Fletcher et al., 1987; Godet & Limpert, 1999; Wolfe et al., 1992). Australian wheat growers began observing a loss of DMI efficacy in 2018 (Lopez‐Ruiz et al., 2023). In the United States, wheat crops generally receive fewer fungicide applications than in the UK and Europe, but regional differences in sensitivity to tebuconazole and prothioconazole were detected in 2013 and 2014 Bgt collections, suggesting some loss of efficacy in eastern states where mildew epidemics are more common (Meyers et al., 2019). Overall, there is a need to better understand the molecular genetic mechanisms behind DMI resistance in *B. graminis*. This knowledge could help to redesign DMI molecules to delay the loss of efficacy of this chemistry in the United States, and perhaps revive its utility for mildew control in Europe and elsewhere, for example by facilitating the design of fungicidal molecules that target different parts of the CYP51 protein. Information about the molecular evolution of resistance can also be applied in fungicide resistance monitoring programmes.

Here, we report the first comprehensive study of the molecular basis of triazole resistance in a large collection of US Bgt isolates from several wheat‐growing regions of the United States, and a smaller collection of UK isolates, with both collections apparently representative of contemporary DMI sensitivity levels. In both samples, we examined *Cyp51B* (hereafter *Cyp51*) for point mutations, gene copy number variation and expression levels in relation to sensitivity to the triazole fungicides tebuconazole and prothioconazole. The international sample comparison elucidated the complex molecular evolution of DMI resistance and provided insights that could help slow its evolution in the US Bgt population.

## 2 RESULTS

### 2.1 Bgt isolates used

Three groups of Bgt isolates were studied: 363 US isolates derived from commercial wheat fields in 2013–2014 (Meyers et al., 2019), of which 30 isolates were studied for copy number per nucleus and expression of *Cyp51*; 46 UK isolates collected from wheat in greenhouses and outdoor locations in Norfolk in 2014–2015; and a set of four older reference isolates collected between 1985 and 1998 in the UK, Germany and Switzerland (Tables 1 and S1). All isolates had been single‐pustuled and subcultured at derivation and were genetically pure.

**TABLE 1:** *Cyp51* alleles in *Blumeria graminis* f. sp. *tritici* isolates collected from commercial fields in the United States and from both glasshouses and fields in the United Kingdom.^a^

Allele	Codon 136	Codon 509	Number (%) of US isolates^b^	Number of UK isolates^c^		Number (name) of reference isolates
				Glasshouse	Field	
Y136+S509 (wild type)	TAT	TCC	227 (62.5%)	0	0	2 (94202, JIW11)
F136+S509	TTT	TCC	103 (28.5%)	22	8	1 (Fel09)
F136+T509	TTT	ACC	0	1	0	0
Het (Y136+S509 and F136+S509)	TAT & TTT	TCC	33 (9.0%)	0	0	0
Het+Het (Y136+S509 and F136+T509)	TAT & TTT	TCC & ACC	0	7	8	1 (96224)
Total			363	30	16	4

^a^ US alleles are differentiated from one another by the sequence at codon 136, while UK alleles are differentiated by the sequences at codons 136 and 509.

^b^ Sampling locations and numbers in Figure 3 and Table S7.

^c^ All collected in Norfolk, UK.

The 30 US isolates studied for copy number and expression represented all 15 states from which the larger isolate collection had originated and all available combinations of three factors: variant at codon 136 and high or low sensitivity to each of the two fungicides. All available US isolates with heteroallelic *Cyp51* were also included. Thus, it was highly unlikely that any of the 30 US isolates were clone mates (Parks et al., 2008).

**FIGURE 1:**
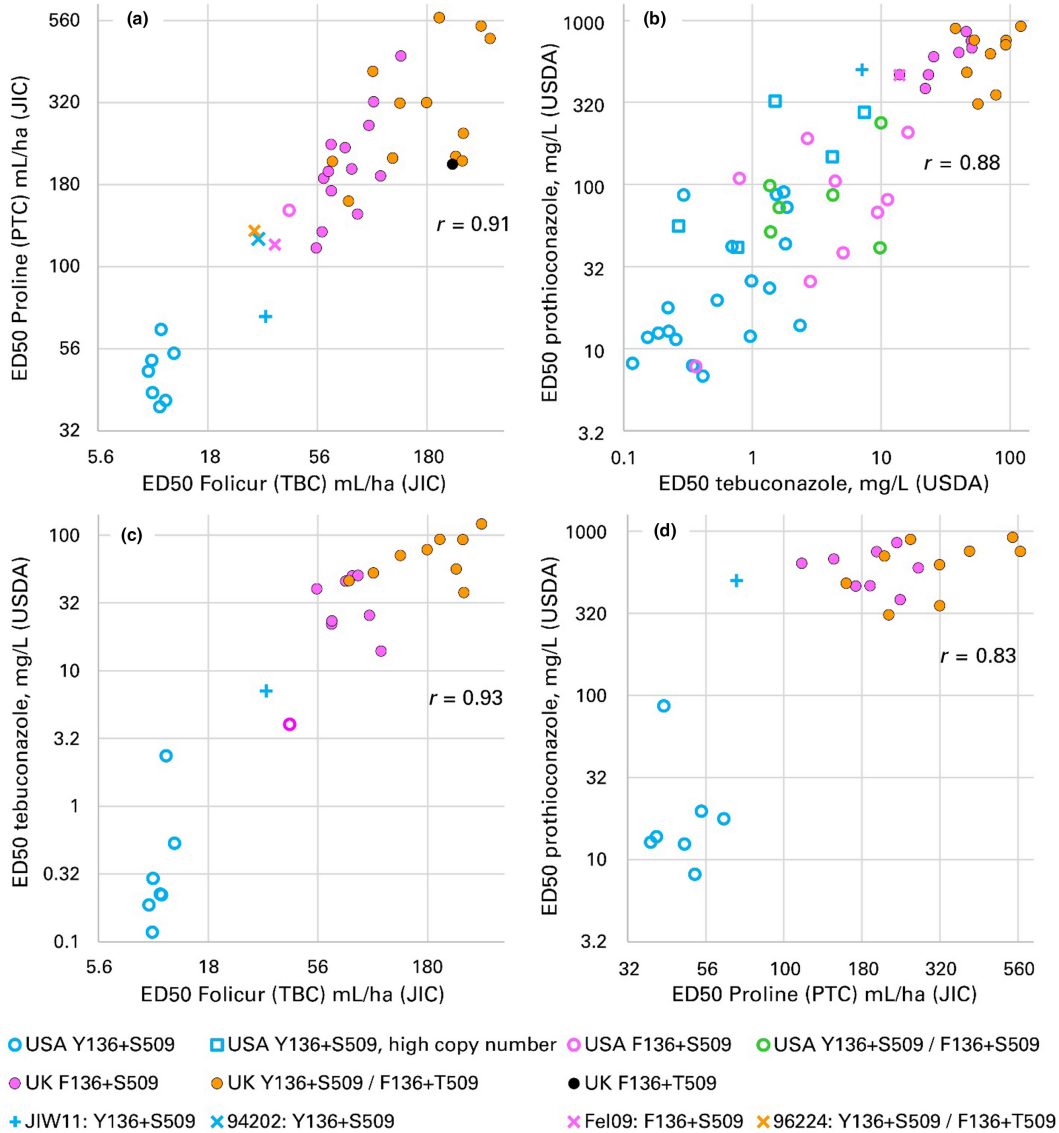
Sterol demethylation inhibitor (DMI) sensitivity (ED_50_) of *Blumeria graminis* f. sp. *tritici* isolates by *Cyp51* genotype; relationships of (a) Proline (prothioconazole, PTC) to Folicur (tebuconazole, TBC) at the JIC, (b) PTC to TBC at USDA, (c) TBC at USDA to Folicur (TBC) at JIC, (d) PTC at USDA to Proline (PTC) at JIC. JIC, John Innes Centre; USDA, U.S. Department of Agriculture‐Agricultural Research Service in Raleigh, North Carolina.

**FIGURE 2:**
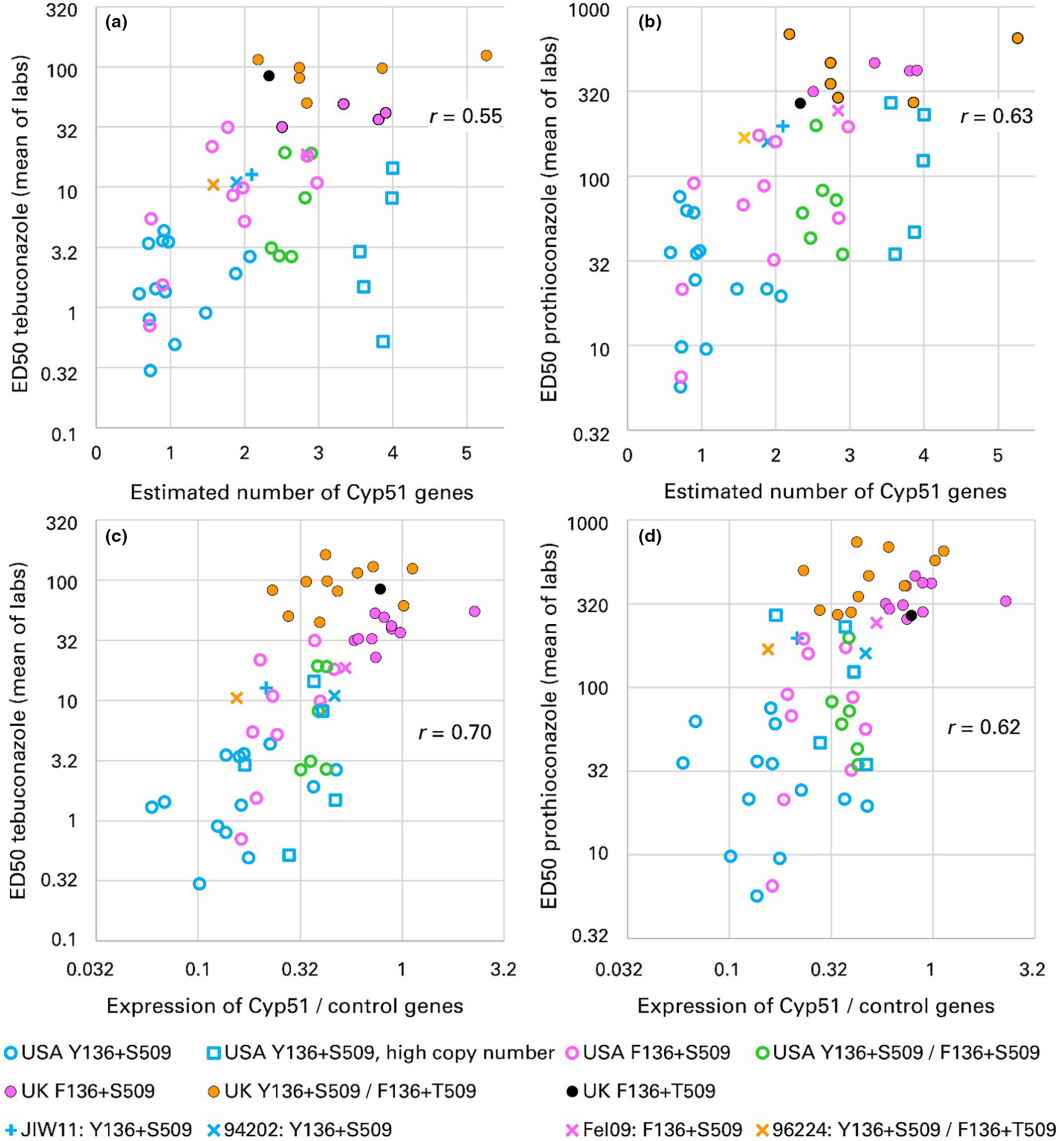
Mean sterol demethylation inhibitor (DMI) sensitivity (ED_50_) across JIC and USDA laboratories of *Blumeria graminis* f. sp. *tritici* isolates by *Cyp51* allele; correlations of (a) tebuconazole ED_50_ with estimated *Cyp51* gene number, (b) prothioconazole ED_50_ with estimated *Cyp51* gene number, (c) tebuconazole ED_50_ with *Cyp51* expression and (d) prothioconazole ED_50_ with *Cyp51* expression. *Cyp51* copy number was estimated by droplet‐digital PCR relative to the single‐copy *Tub2* gene. *Cyp51* expression was measured by reverse transcription‐quantitative PCR. JIC, John Innes Centre; USDA, U.S. Department of Agriculture‐Agricultural Research Service in Raleigh, North Carolina. Within a panel, the same letter above boxes indicates means are not different at *p* < 0.05.

Variation in the UK isolates was characterized by simple‐sequence repeat (SSR) variation and mating type. Many, but not all, glasshouse isolates were one of four genotypes, within which all isolates had identical SSR markers, mating types and virulence (Tables S1 and S2). As Bgt has a mixed reproductive system with an approximately annual sexual cycle (Parks et al., 2009), it is likely that these genotypes, named lineages 1, 2, 3 and 5, each represent a distinct clone of the fungus. In statistical models of *Cyp51* copy number and gene expression by reverse transcription‐quantitative PCR (RT‐qPCR), variation between isolates within lineages was small (Tables S3 and S4). Each glasshouse lineage is therefore treated as one genotype for the purpose of discussing factors associated with triazole resistance, represented by one point in each graph in Figures 1 and 2, and treated as one lineage in Tables S3 and S4. All remaining glasshouse isolates and all isolates from the natural air spora differed from each other and from the four named lineages in one or more characteristics, and thus represented different clones.

### 2.2 Azole sensitivity of US and UK isolates

Tebuconazole and prothioconazole sensitivities of all isolates were assayed, along with characteristics related to *Cyp51*. While prothioconazole itself is a triazolinethione, its primary breakdown metabolite in planta is the triazole prothioconazole‐desthio (PD), which is the active fungicide (Parker et al., 2013). In our tests, we used prothioconazole, not PD, because the fungicide was applied to the plant and thus indirectly to Bgt on the leaf.

Responses of Bgt isolates to tebuconazole and prothioconazole expressed as ED_50_ values were highly correlated, both with the precision spray method used at the John Innes Centre (JIC) (Figure 1a) and the spray to run‐off method in North Carolina (Figure 1b). Despite the different methods of fungicide application used by the UK and US researchers, logED_50_ values estimated in the two laboratories were highly correlated for each fungicide (Figure 1c,d).

**FIGURE 3:**
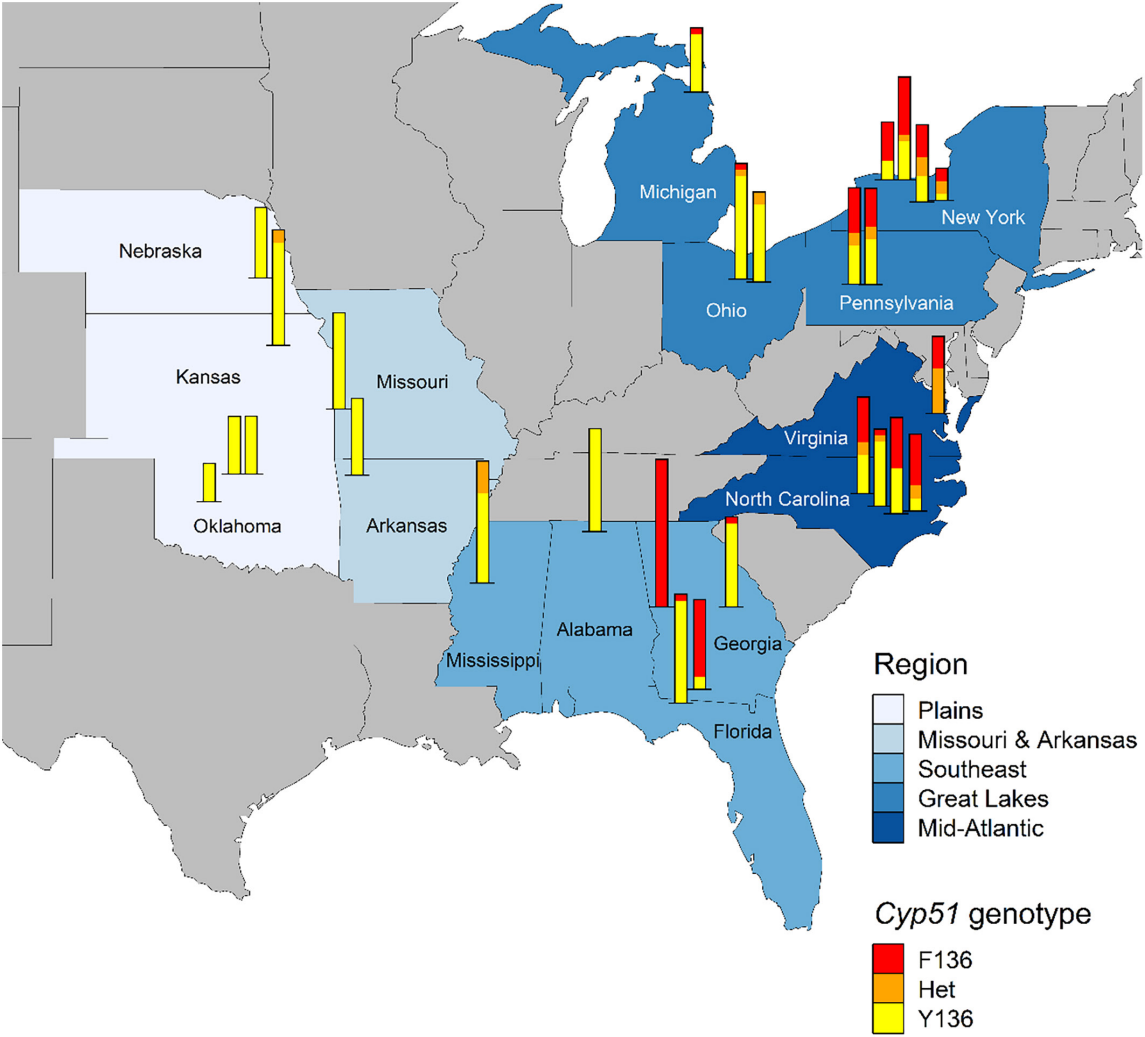
Geographic distribution of *Cyp51* genotypes in the US *Blumeria graminis* f. sp. *tritici* (Bgt) collection from 2013 and 2014. The *Cyp51* genotypes of a total of 363 isolates are shown, with each stacked bar representing the sample from one of 27 fields, and bar heights proportional to sample sizes. Y136 is wild type, F136 is mutant, and Het (heteroallelic) indicates the presence of both alleles in an isolate. All US isolates have S509. Darker blue shading of regions indicates generally higher exposure of Bgt to sterol demethylation inhibitor (DMI) fungicides and reduced sensitivity to tebuconazole and prothioconazole (Meyers et al., 2019).

Overall, US isolates were much more sensitive to tebuconazole and prothioconazole than UK isolates (Figure 1c,d). The ED_50_ values of the full set of 363 US Bgt isolates had been previously determined (Meyers et al., 2019). Briefly, among all isolates tested previously in North Carolina, there was a 155‐fold range in ED_50_ values of tebuconazole and a 1556‐fold range for prothioconazole. Among the US isolates in the present paper, ED_50_ of tebuconazole ranged from 0.12 to 16 mg L^−1^ with a mean of 1.3 mg L^−1^, a 138‐fold range (Table S1). Prothioconazole was less active against US Bgt isolates, with a 355‐fold range in ED_50_ from 6.8 to 322 mg L^−1^ (mean 43 mg L^−1^; Figure 1b). There were significant regional differences in DMI sensitivity in the United States, with eastern subpopulations displaying greater resistance than central US subpopulations (Figure 3). This reflects regional variation in applications of triazole fungicides to wheat (Meyers et al., 2019).

Bgt isolates from the UK sampled in 2014 and 2015 were more resistant to both tebuconazole and prothioconazole than almost all US isolates (Table S1; *t* tests: *p* < 0.001 in both cases; Figure 1c,d). The mean ED_50_ of UK isolates was 17 times higher than that of US isolates for tebuconazole and 8.7 times higher for prothioconazole, and there was little overlap between the responses of UK and US isolates to either tebuconazole or prothioconazole. UK isolates had less variation than US isolates in their responses to triazoles, implying that they had relatively uniform resistance to these fungicides. Averaging across test methods, the UK isolates had a 7.6‐fold range in ED_50_ values of tebuconazole and 4.4‐fold for prothioconazole, whereas the US isolates' responses spanned a 105‐fold and 47‐fold range for tebuconazole and prothioconazole, respectively. The reference isolates Fel09 and JIW11 were used in both the US and UK laboratories, in both of which the ED_50_ values of both fungicides for both isolates were above the median sensitivity of US isolates and below that of UK test isolates (Figure 1a,b).

### 2.3 Mutations in the *Cyp51* gene sequence

A limited range of variation among *Cyp51* alleles was detected in the US and UK populations (Table 1); alleles are named according to the amino acids at residues 136 and 509. In the United States, the full open reading frame (ORF) of *Cyp51* was sequenced in 363 Bgt isolates, revealing variation at codon 136 [137]. Wild‐type Y136 (codon TAT; Y136+S509 allele) and mutant F136 (TTT; F136+S509) were found in 62.5% and 28.5% of the US 363‐isolate collection, respectively, while the remaining 9% of the samples were heteroallelic with both these alleles (Het+S509; discussed further below). All US isolates had wild‐type S509 [S524]. The genotypes of modern UK isolates were F136+S509, F136+T509 and Het+Het (Y136+S509 and F136+T509); none had Y136 only (Table 1). Het+Het isolates were identified as heteroallelic because in sequences of single *Cyp51* genes from isolates with both variants, F136 was invariably associated with T509 and Y136 with S509. All US isolates had the mutation K175N relative to reference isolate 96224, but all UK isolates had K175. Moreover, US but not UK isolates had a single‐nucleotide polymorphism (SNP) in *Cyp51* intron 2. No nonsynonymous variation at codons other than 136 or 509 was detected in UK *Cyp51* sequences. There was no variation in either US or UK isolates at codons 171, 301 and 327, where polymorphism has been reported in Bgh from Western Australia (Tucker et al., 2019).

Among the older European reference isolates, 94202 and JIW11 were Y136+S509 and Fel09 was F136+S509. 96224 was Het+Het, with both Y136+S509 and F136+T509 *Cyp51* alleles. Note that the *Cyp51* gene Bgt*A‐21131* in the 96224 assembly v3.16 has the F136 codon (TTT), while Bgt*A‐21131* on Scaffold 60 in the older 2013 assembly has the Y136 codon (TAT) (GenBank: EPQ62754.1, NCBI: BGT96224_A21131).

### 2.4 Relationship of DMI sensitivity to *Cyp51* gene sequence

**FIGURE 4:**
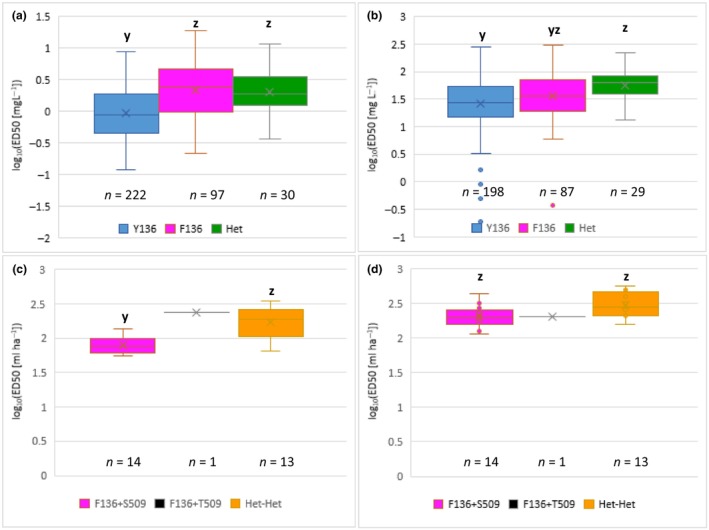
Sensitivity to sterol demethylation inhibitor (DMI) fungicides tebuconazole and prothioconazole of *Blumeria graminis* f. sp. *tritici* isolates collected in the United States and the UK in 2013–2015. (a) Tebuconazole ED_50_ values of US isolates, (b) prothioconazole ED_50_ values of US isolates, (c) tebuconazole ED_50_ values of UK isolates and (d) prothioconazole ED_50_ values of UK isolates. Genotypes: Y136 = wild type; F136 = Y136F substitution; Het = heteroallelic, Y136‐S509/F136‐S509 (only found in the United States); and Het‐Het = Y136‐S509/F136‐T509 (only found in the UK). *p* values indicate significance of differences among means; within a graph, boxes topped by the same letter indicate means are not different at *p* < 0.05.

The isolates tested were divided into six groups by genotype and country of origin to investigate the relationship of fungicide ED_50_ value to *Cyp51* allele: Y136+S509, F136+S509 and Het+S509 in the United States, and F136+S509, F136+T509 and Het+Het in the UK. Reflecting the high correlation between responses to the two fungicides, there was a strong association between groups and mean ED_50_ values of both triazoles (Figure 4; Table S5; Group term in Table S6: *p* < 0.001), but there was also significant variation between groups' responses to the two fungicides separately (Fungicide: Group term in Table S6; *p* < 0.001). Although the numerical range of responses differed between the two laboratories, their methods produced largely consistent results (Lab:Fungicide:Group term in Table S6; *p* = 0.3). Groups were therefore compared by combined data from both laboratories, using means and standard errors from the Fungicide:Group term.

US Y+S wild‐type isolates were highly sensitive on average to both tebuconazole (Folicur) and prothioconazole (Proline). In tests at JIC, they were more sensitive to both fungicides than all four reference isolates, including two with Y+S, 94202 and JIW11 (Figure 1a). In the USDA tests, all US Y136+S509 isolates but one were more sensitive to tebuconazole than JIW11 and all were more sensitive to prothioconazole than JIW11 (Figure 1b). US Y136+S509 isolates were significantly more sensitive than US F136+S509 isolates to tebuconazole (Figure 4a; resistance factor [RF] as ratio of means of less sensitive and more sensitive groups = 3.7; *p* = 0.002; *t* tests in Table S6d); although they had lower mean ED_50_ values to prothioconazole than US F136+S509 isolates (Figure 4b), the difference was not statistically significant (RF = 1.7; *p* = 0.2). US Het+S509 isolates were significantly more resistant to both tebuconazole (RF = 4.7; *p* < 0.001) and prothioconazole (RF = 2.6; *p* = 0.03) than US Y136+S509 isolates but did not differ significantly from US F136+S509 isolates in their sensitivity to either azole (RF = 1.1, *p* = 0.9 for tebuconazole; RF = 1.3, *p* = 0.6 for prothioconazole; Figure 4a,b).

Among UK isolates, Het+Het isolates were significantly less sensitive to tebuconazole than F136+S509 isolates (RF = 2.1; *p* = 0.02) but had similar responses to prothioconazole (Figure 4c,d; RF = 1.2; *p* = 0.5). UK F136+S509 isolates were more resistant than US F136+S509 isolates to both tebuconazole (RF = 3.9; *p* = 0.002) and prothioconazole (RF = 4.1; *p* = 0.001). Only one F136+T509 isolate was obtained, which had an ED_50_ of tebuconazole substantially higher than UK F136+S509 isolates (RF = 3.0; *p* = 0.009) but similar to UK Het+Het isolates (RF = 1.4; *p* = 0.4). It had a similar response to prothioconazole as the UK F136+S509 (RF = 1.0; *p* = 1.0) and Het+Het isolates (RF = 1.5; *p* = 0.3).

### 2.5 US geographic distribution of *Cyp51* alleles

In the United States, where T509 was not detected, the F136+S509 allele was particularly common in the Atlantic seaboard states (Figure 3), where previous findings on reduced triazole sensitivity and national fungicide application data had suggested greater exposure of Bgt to triazoles (Meyers et al., 2019). By contrast, the Plains and adjacent Arkansas‐Missouri samples were dominated by wild‐type Y136+S509 isolates. In those central US states, where greater Bgt sensitivity to tebuconazole and prothioconazole had been observed, mildew epidemics are less frequent and fungicide applications to wheat are less consistent. Although two fields in Georgia had high F136+S509 frequencies, isolates from the south‐east region overall were predominantly Y136+S509, as were the majority of Michigan and Ohio isolates.

The Het+S509 genotype was widely distributed in the Mid‐Atlantic and Great Lakes regions (Figure 3), which were previously found to constitute a single random‐mating population (Cowger et al., 2016). The greatest *Cyp51* allelic diversity was found in North Carolina, New York and Pennsylvania, where all three genotypes were usually found in each field. In addition, Het+S509 strains were identified in one field each in Kansas and Mississippi, remote from the Mid‐Atlantic and Great Lakes regions.

### 2.6 *Cyp51* copy number

*Cyp51* copy number per nucleus, relative to the single‐copy gene *Tub2*, was estimated by droplet‐digital PCR (ddPCR) (Table 2; Figure 2a,b) and ranged from 0.6 to 4.0 (mean 1.9) in the United States and from 2.2 to 5.3 (mean 3.2) in the UK, excluding reference isolates. The difference between the two countries in mean gene number was highly significant (*t* test, *p* = 0.001; Table 2). There was significant variation between the six genotype‐country groups defined above (*F* = 3.36, *df* = 5 and 40, *p* = 0.01; Table S3). Within the United States, Y136+S509 and F136+S509 isolates had similar mean numbers of *Cyp51* genes (*p* = 0.8), while Het+S509 isolates had more *Cyp51* genes than either homoallelic class, although the difference was slight (*p* = 0.1 for each comparison). A striking feature of the US Y136+S509 isolates was that five of them had much higher estimated *Cyp51* numbers per nucleus (3.6–4.0) than the other 13 isolates (0.6–2.1; Figure 2a,b). In the UK, F136+S509 and Het+Het isolates had similar mean numbers of *Cyp51* genes (*p* = 0.9; Table 2). The F136+T509 isolate had fewer *Cyp51* genes than either the F136+S509 or Het+Het groups. UK F136+S509 isolates had significantly more *Cyp51* genes than US F136+S509 isolates (*p* = 0.01).

**TABLE 2:** Mean *Cyp51* copy number by genotype and origin for *Blumeria graminis* f. sp. *tritici* isolates collected in 2013–2014 in the US and 2014–2015 in the UK.^a^

Genotype	Origin		Reference isolates^b^			
	US^c^	UK^d^	JIW11	94202	Fel09	96224
Y136	1.8	–	2.1	1.9		
F136+S509	1.7	3.8			2.8	
F136+T509	–	2.3				
Het	2.6	–				
Het+Het	–	3.3				1.6
Overall^e^	1.9 y	3.2 z				

^a^ As determined by droplet‐digital PCR.

^b^ JIW11 was collected in the UK in 1985 and 94202 in Switzerland in 1994. Fel09 was collected in Germany ~1998 and 96224 in Switzerland, 2006.

^c^ 34 US isolates collected from 15 states.

^d^ 11 UK isolates and clonal lineages.

^e^ Across genotypes, the US‐UK difference was significant (*p* = 0.001), indicated by different letters.

Both European Y136+S509 reference isolates, 94202 and JIW11, had more copies of *Cyp51* than almost all the larger group of US Y136+S509 isolates with low *Cyp51* copy numbers but fewer than the five with high copy numbers (Figure 2a,b). The F136+S509 reference Fel09 had a higher *Cyp51* copy number than almost all US F136+S509 isolates and the UK F136+T509 isolate but fewer copies than all but one UK F136+S509 isolate (Figure 2a,b). The Het+Het reference 96224 had a similar gene number as the mean of US F136+S509 isolates but fewer than either the UK Het+Het or US Het+S509 isolates.

### 2.7 *Cyp51* gene expression

**FIGURE 5:**
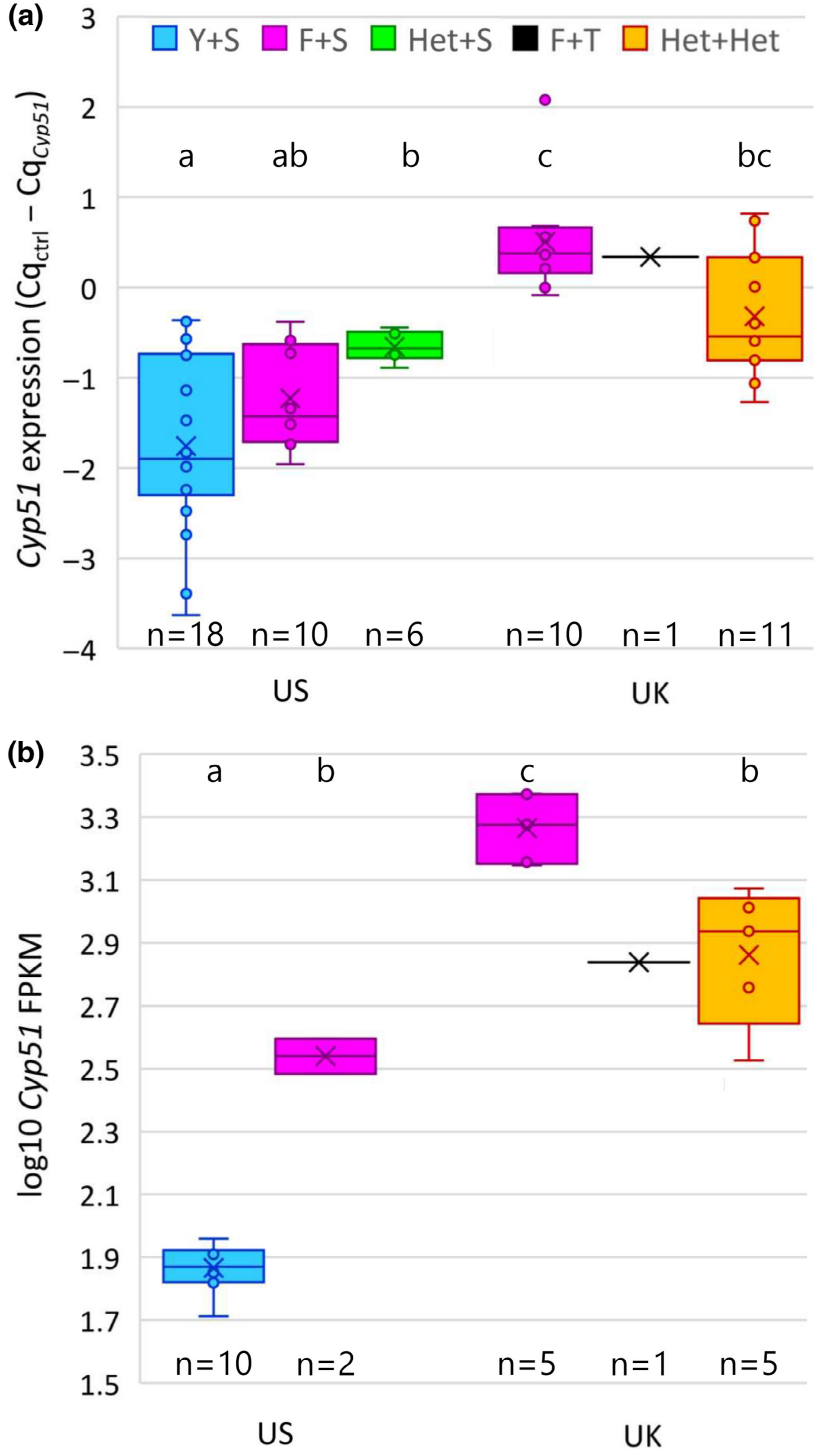
*Cyp51* expression in *Blumeria graminis* f. sp. *tritici* isolates and lineages from the UK and the United States as determined by (a) reverse transcription‐quantitative PCR (RT‐qPCR) and (b) RNA‐seq, where FPKM = fragments per kilobase million. *Cyp51* alleles: Y136 = wild type, F136 = mutant (Y136F), Het = Y136+S509 and F136+S509 (only tested by RT‐qPCR), and Het‐Het = Y136+S509 and F136+T509.

Mean expression of *Cyp51* relative to standard control genes estimated by RT‐qPCR was 2.7 times higher in UK isolates than in US isolates (*t* = 6.71, *df* = 54, *p* < 0.001; Table 3, Figures 2c,d and 5). There was highly significant variation between the six groups defined above (*F* = 14.63, *df* = 5 and 50, *p* < 0.001). US F136+S509 isolates had higher mean *Cyp51* expression than US Y136+S509 isolates, but the difference was not statistically significant (*p* = 0.08). US Het+S509 isolates had mean expression higher than either the homoallelic US Y136+S509 (*p* = 0.004) or US F136+S509 isolates, although the difference between Het+S509 and F136+S509 was not significant (*p* = 0.2). In the UK, *Cyp51* expression was 1.7 times higher in F136+S509 isolates than Het+Het isolates (*p* = 0.01) and 1.6 times higher in the F136+T509 isolate than in Het+Het isolates. Among F136+S509 isolates, expression was 3.2 times higher in UK than US isolates (*p* < 0.001).

**TABLE 3:** Mean *Cyp51* expression by genotype and origin for *Blumeria graminis* f. sp. *tritici* isolates collected in 2013–2014 in the United States and 2014–2015 in the UK.^a^

Genotype	Origin		Reference isolates^b^			
	US^c^	UK^d^	JIW11	94202	Fel09	96224
Y136	0.19	–	0.22	0.47		
F136+S509	0.27	0.85			0.53	
F136+T509	–	0.78				
Het	0.38	–				
Het+Het	–	0.49				0.15
Overall^e^	0.24 y	0.64 z				

^a^ As determined by reverse transcription‐quantitative PCR.

^b^ JIW11 was collected in the UK in 1985 and 94202 in Switzerland in 1994. Fel09 was collected in Germany ~1998 and 96224 in Switzerland, 2006.

^c^ 34 US isolates collected from 15 states.

^d^ 22 UK isolates and clonal lineages.

^e^ Across genotypes, the US‐UK difference was highly significant (*p* < 0.001), indicated by different letters.

Considering the reference strains, *Cyp51* expression was lower in almost all US Y136+S509 isolates than in the European Y136+S509 reference isolate 94202 but comparable to the older reference, JIW11 (Table 3). In the F136+S509 reference Fel09, the expression was higher than all US F136+S509 isolates but lower than all the more recent UK F136+S509 and F136+T509 isolates. Similarly, the Het+Het reference 96224 had lower expression than all other Het+Het isolates.

**FIGURE 6:**
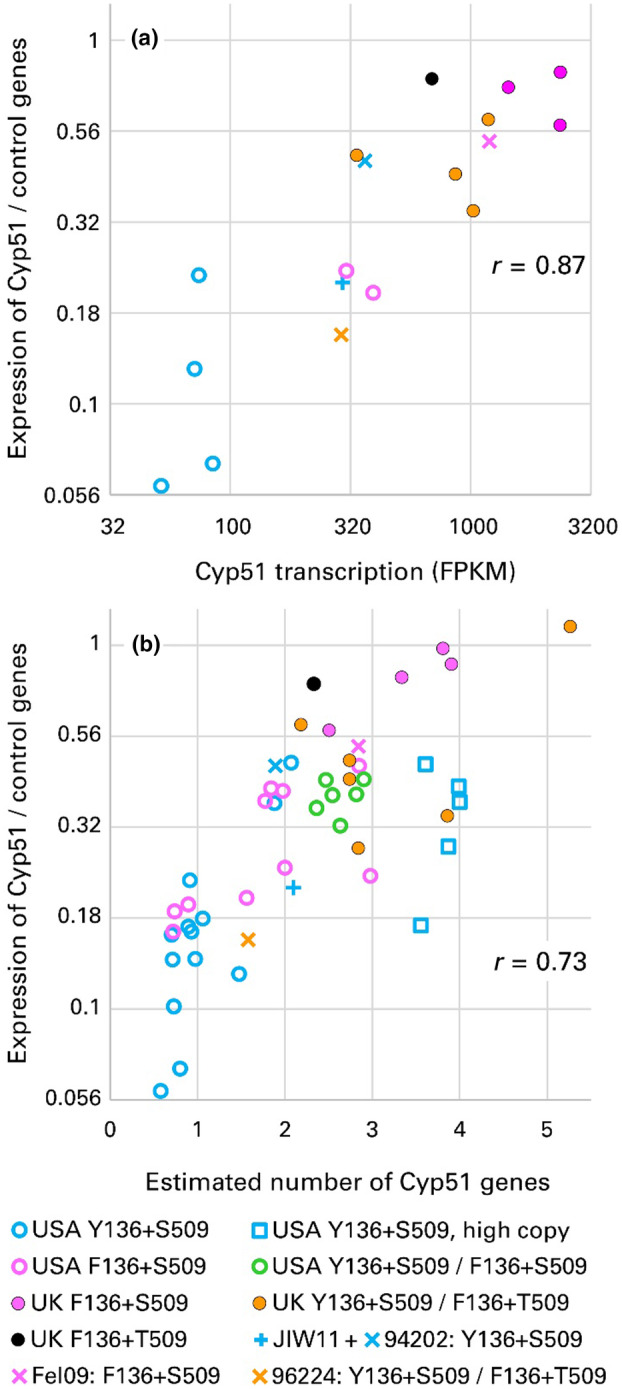
(a) Correlation of *Cyp51* expression in *Blumeria graminis* f. sp. *tritici* (Bgt) measured by reverse transcription‐quantitative PCR (RT‐qPCR) and by fragments per kilobase of transcript per million mapped reads (FPKM) in RNA‐seq. Bgt isolates classified by country of origin and predicted amino acids at residues 136 and 509. Fel09, JIW111, 94202, 96224: Reference isolates. A group of US Y136+S509 isolates with high copy number of *Cyp51* are marked separately. *r*: Pearson correlation coefficient. (b) Correlation of the estimated number of *Cyp51* genes and *Cyp51* expression. *Cyp51* copy number was estimated by droplet‐digital PCR relative to the single‐copy *Tub2* gene. *Cyp51* expression was measured by RT‐qPCR; values displayed are expression relative to the mean of three reference genes, *actin*, *GAPDH* and *Tub2*.

*Cyp51* expression estimated by RT‐qPCR was highly correlated with the proportion of *Cyp51* in mRNA as estimated by RNA‐seq (Figure 6a, *p* < 0.001). In terms of fragments per kilobase of transcript per million mapped reads (FPKM), an average of 4.8‐fold upregulation of *Cyp51* was apparent in US F136+S509 isolates compared to US Y136+S509 isolates (*t* test of log_10_FPKM; *p* < 0.001). The US F136+S509 isolates showed similar levels of expression to the European Y136+S509 reference isolates JIW11 and 94202, using RNA‐seq data (Figure 6a). UK F136+S509 isolates had the highest rate of *Cyp51* transcription, with mean FPKM 5.3 times higher than US F136+S509 isolates and 25 times higher than US Y136+S509 isolates (*p* < 0.001 in both cases). They also had higher FPKM than the UK F136+T509 isolate (2.7‐fold; *p* = 0.007) and the UK Het+Het isolates (2.5‐fold; *p* < 0.001). All seven Het+Het isolates for which transcription was studied expressed both the Tyr136 and Phe136 alleles, with the proportion of the Phe136 codon in the mRNA (AAA) being 73% in 96224 and ranging from 82% to 93% in UK field and glasshouse isolates.

There was a strong correlation between *Cyp51* copy number per nucleus and gene expression measured by either method (Figure 6b; *p* < 0.001 for correlation of copy number with either method of estimating *Cyp51* expression; correlation coefficient of copy number with FPKM = 0.80). The five US Y136+S509 isolates with high gene numbers expressed *Cyp51* at a level somewhat lower than would be predicted from their gene number but nonetheless more highly than the majority of US Y136+S509 isolates, comparable to US F136+S509 and Het isolates. Both *Cyp51* expression and *Cyp51* gene number were strongly correlated with resistance to both azole fungicides (Figure 2, *p* < 0.001, for all four correlations).

### 2.8 Genetic associations with azole resistance

**FIGURE 7:**
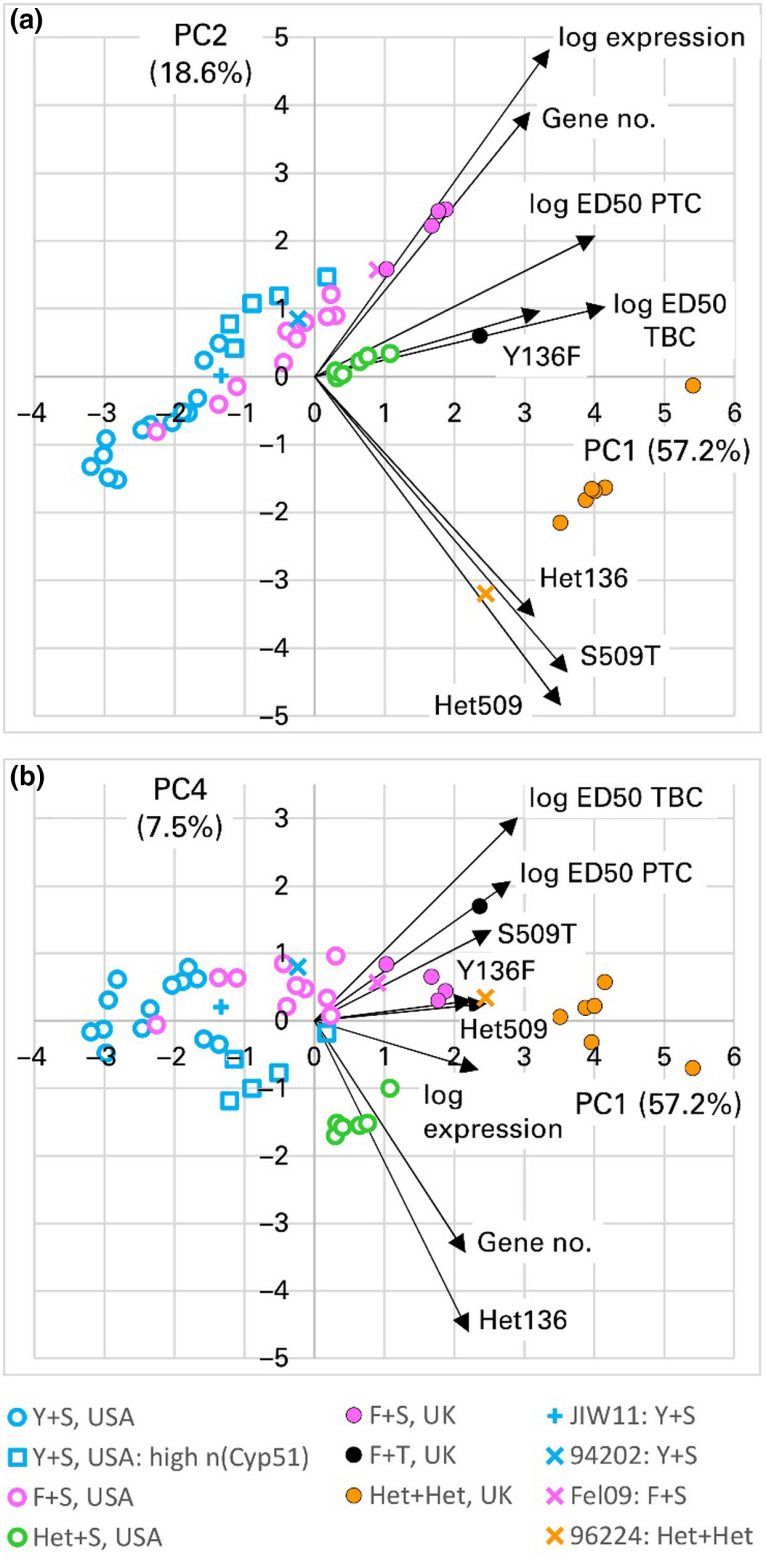
Principal component (PC) analysis of responses of *Blumeria graminis* f. sp. *tritici* (Bgt) isolates to triazole fungicides, predicted CYP51 protein sequence variants and copy number and expression of *Cyp51*. Biplots of (a) PCs 1 and 2 and (b) PCs 1 and 4. ED_50_ values of tebuconazole and prothioconazole and expression of *Cyp51* relative to the mean of three reference genes, *actin*, *GAPDH* and *Tub2*, were measured by reverse transcription‐quantitative PCR and log_10_‐transformed. The length of the biplot axis for each variable is proportional to its loading on each PC. Bgt isolates classified by country of origin and predicted amino acids at residues 136 and 509. JIW11, 94202, Fel09, 96224: Reference isolates. A group of US Y136+S509 isolates with high copy number of *Cyp51* is marked separately.

All factors studied, including both sequence variants (Y136F and S509T), heteroallelism at both mutated residues, the number of *Cyp51* genes per nucleus, and their expression were all correlated with one another and with resistances to each azole (Figures 2 and 6). This makes it difficult to separate the effects of each genetic variable, but insights into their relationship with azole resistance can be gained by multivariate analysis. Principal component analysis (PCA) was done for the 49 isolates that were scored for all these traits (Figure 7).

All variables had positive loadings on the first principal component (PC1), which accounted for 57% of variation. This reflected the positive correlations between fungicide resistance and all the other variables, given that loadings are the contributions that each of the original variables makes to a PC. The second principal component (PC2), including 19% of variation, largely contrasted changes associated with stronger resistance (S509T and heteroallelism), which had negative loadings, against increased gene number and expression, which had positive loadings. These relationships are apparent in Figure 7a, where isolates with low PC1 values, particularly the US Y136+S509 group, had Y136 and S509, low *Cyp51* copy number, low *Cyp51* expression and low ED_50_ values of tebuconazole and prothioconazole.

Isolates with higher values of PC1 showed two trends. One was that UK F136+S509 isolates and to a lesser extent, the US F136+S509 group had, in addition to F136, higher azole resistance than most Y136 isolates, and higher copy number per nucleus and expression of *Cyp51*. The second trend was that the Het+Het group had even higher resistance associated with the S509T substitution and heteroallelism at both the critical residues. High *Cyp51* copy number and expression contributed to resistance but so did the additional sequence variants. This was reflected in the distribution of the Het+Het isolates in the biplot (Figure 7a); note that CAW15S6341 (with coordinates 5.41, −0.13) had the highest estimated gene number of all the isolates and among the highest *Cyp51* expression and ED_50_ values of both azoles, whereas 96224 (coordinates 2.45, −3.19) had lower gene number, expression and ED_50_ values. The homoallelic F136+T509 isolate had high resistance resulting from the T509 residue and high *Cyp51* expression although its *Cyp51* copy number was lower than the mean of both the UK F136+S509 and Het+Het group. The US Het+S509 group had higher resistance than the US F136+S509 isolates as a result of heteroallelism at residue 136, despite *Cyp51* copy number and expression not necessarily being higher than the US F136+S509 group (Figures 5 and 6).

Gene number and *Cyp51* expression were closely correlated among most isolates and made similar contributions to PC1 and PC2, which together accounted for 76% of variation. However, gene number made a large contribution to PC4 but gene expression did not (Figure 7b). Although PC4 only accounted for 8% of variation, the five US Y+S isolates with high copy number were distinct from the majority of the US Y+S group, being shifted in the direction of higher values on the copy number axis (PC3 is not included in Figure 7 because it had little contribution from ED_50_ values of either azole and thus had a low correlation with azole resistance).

### 2.9 Sporulation variation by *Cyp51* genotype

Sporulation, a component of isolate fitness, was measured for 351 of the US Bgt isolates. Mean sporulation of F136+S509 isolates was 19% lower than Het+S509 isolates and 16% lower than Y136+S509 (Figure S1). The wide variation between isolates of each genotype meant that even these large differences were not statistically significant (*p* = 0.2).

## 3 DISCUSSION

This study is a comprehensive examination of the molecular basis of triazole resistance in Bgt, a model organism among the powdery mildews. *Cyp51* encodes the target enzyme of triazoles, one of the largest classes of fungicide used to control fungal pathogens of crops, farm animals and humans worldwide.

We found only two amino acid substitutions, Y136F [Y137F] and S509T [S524T] associated with triazole resistance in hundreds of geographically diverse isolates, and they could not explain the wide variation in resistance between the United States and the UK, within the United States, or between recent and older UK isolates. We therefore investigated two other forms of genetic variation: *Cyp51* copy number per nucleus using ddPCR and *Cyp51* gene expression with both RT‐qPCR and RNA‐seq. We propose a genetic model relevant to all our isolates—US and UK isolates, and older and newer isolates from Europe—in which gene sequence, copy number and expression combine to determine the response to triazoles. Across our isolate collection, copy number and gene expression were correlated, which was not surprising. What was novel, however, was that mean copy number was significantly higher in the UK than the United States; this demonstrated one of two mechanisms by which the UK isolates have progressed significantly further along the path to triazole resistance than their US counterparts.

UK Bgt isolates sampled in 2014 and 2015 were much more resistant to triazole fungicides than even the most resistant contemporary US Bgt isolates. Estimation of ED_50_ values of both prothioconazole and tebuconazole for UK test isolates required an expanded range of experimental fungicide concentrations relative to the standard method used in the USDA laboratory. In these tests, the maximum observed ED_50_ value of US isolates was near that of the minimum observed UK value. This implies that there has been much weaker selection for azole resistance in the United States, which has had much lower per‐hectare application of fungicides to wheat than the UK.

Responses to the two azoles were strongly but not completely correlated and four genetic mechanisms were associated with greater resistance to these fungicides (Figures 1 and 2). One is the amino acid sequence of the CYP51 protein, involving F136 and T509 substitutions, and another is heteroallelism involving wild‐type and mutant alleles of *Cyp51*. A third is an increase in both copy number and expression of *Cyp51*, associated with higher resistance to both azoles in both homoallelic and heteroallelic isolates. Finally, a group of US Y136+S509 isolates had substantially more copies of wild‐type *Cyp51* than the majority of Y136+S509 isolates and had enhanced resistance to prothioconazole but less so or not at all to tebuconazole.

A striking feature of azole resistance in *B. graminis* is that very few changes in the CYP51 protein other than F136 and T509 are involved. A third CYP51 substitution, K175N, was previously detected in both azole‐resistant and azole‐sensitive Bgt isolates in the UK (Wyand & Brown, 2005), but has now been shown not to be associated with azole responses because all US isolates had N175. In Bgh in Western Australia, only three additional changes were detected in CYP51—K171E [R172 in *Z. tritici*], M301I [M304I] and R327G [R330G]—but none of them increased resistance significantly above the level achieved by F136 or F136+T509 (Tucker et al., 2019). In the UK, a clonal group of Bgh isolates with very high resistance to azoles had a K147Q substitution [K148Q] in addition to F136 (Wyand & Brown, 2005). This change has not been reported in other powdery mildew isolates, but it is plausible that it is involved in resistance because K143R combined with Y132F at homologous residues in *Candida albicans* was associated with resistance to fluconazole (Warrilow et al., 2019). None of the substitutions identified here, other than F136 and T509, are at sites likely to affect fungicide activity. The limited variation in Bgt is consistent with other powdery mildews including *E. necator* in the United States with substitutions only at residue 136 (Rallos & Baudoin, 2016) and *Podosphaera xanthii* (powdery mildew of cucurbits) with only four substitutions in isolates with high DMI resistance (Ishii et al., 2021). This is a striking contrast to other well‐studied pathogenic fungi such as *Zymoseptoria* (Cools & Fraaije, 2013), *Fusarium* (Vermeulen et al., 2022) and *Aspergillus* (Pérez‐Cantero et al., 2020), which have many more substitutions associated with resistance and, unlike Bgt, are necrotrophs or hemibiotrophs. We speculate that the obligate biotrophic lifestyle of Bgt may constrain its evolutionary ability to vary the tertiary structure of the CYP51 protein and thus its amino acid sequence without a significant detriment to fitness.

Our survey also revealed the widespread presence of a further important mechanism of high resistance, *Cyp51* heteroallelism among genetically pure isolates that have more than one allele of the *Cyp51B* gene. In the United States, Het+S isolates (Y136+S509/F136+S509) had greater resistance to both fungicides than Y136+S509 isolates, similar to that of the F136+S509 group. In the UK, Het+Het isolates (Y136+S509/F136+T509) had significantly greater resistance than the F136+S509 group to tebuconazole but not prothioconazole. Although the present data do not indicate if both alleles are present within a nucleus as a gene duplication, or in different nuclei with heteroallelic isolates being heterokaryotic, or if there is merodiploidy, the first scenario seems tentatively more likely because, unlike the phenomenon of heteroresistance to azoles in *Cryptococcus neoformans* (Chang et al., 2018), heteroallelism in Bgt is stable as the isolates have retained this genotype over years of repeated culturing on detached leaves not treated with fungicide, in the case of 96224 more than 100 subcultures over 27 years.

As in previous studies on powdery mildews (Délye et al., 1998; Tucker et al., 2019; Wyand & Brown, 2005), the F136 substitution was associated with azole resistance but more strongly with resistance to tebuconazole than prothioconazole. Greater resistance to tebuconazole but not prothioconazole was also associated with the combination of the T509 and F136 substitutions in Het+Het and F136+T509 isolates but as in Bgh in Western Australia (Tucker et al., 2019), T509 was never present in combination with Y136 in homoallelic isolates. However, the fact that *Cyp51* heteroallelism conferred greater tebuconazole resistance beyond that provided by the F136 substitution alone suggests that in this UK Bgt sample, as for Bgh in Western Australia, the T509 substitution is specifically conducive to resistance to tebuconazole but not prothioconazole. Supporting this point, the one UK F136+T509 isolate had higher tebuconazole resistance than Het+Het isolates but lower prothioconazole resistance; of course, other F136+T509 isolates would be required to test the relationship between heteroallelism and relative sensitivity to different azoles.

Within the complete set of Bgt isolates and also within the F136+S509 group and the majority of the Y136+S509 isolates, resistance to both azoles was correlated both with larger numbers of *Cyp51* genes per nucleus and with higher *Cyp51* expression. This correlation suggests that for the most part, higher gene number increases resistance by producing greater total expression of *Cyp51*. There are at least two ways in which higher gene expression (and thus more *Cyp51* genes) may be adaptive in azole‐resistant Bgt. Illustrating the first way, it was found that in *Saccharomyces cerevisiae* yeast transformed with *Cyp51* from *Z. tritici*, Y137F conferred partial resistance to azoles but also reduced CYP51 activity (Cools et al., 2011). In homoallelic Bgt isolates, higher *Cyp51* expression in F136+S509 isolates than in Y136+S509 isolates may be either an additional mechanism of azole resistance over and above the amino acid substitutions or a means of compensating for lower activity of CYP51 containing F136. If the F136 variant has lower activity than Y136 in demethylating lanosterol, more of it may be required to maintain flux through the sterol biosynthesis pathway.

Heteroallelism would thus be adaptive because in the presence of the fungicide, the mutant F136 protein could act as the functional variant while the azole‐sensitive wild‐type variant sequestered azole molecules. This would enable the fungus to overcome CYP51 inhibition by the azole while maintaining overall expression near that of the wild type and retaining near‐normal demethylation activity. In the absence of an azole fungicide, for example, in early host growth stages or in the off season, the wild‐type Y136+S509 variant would have higher activity than the mutated F136+S509 or F136+T509 protein. This sequestration model for mitigating a fitness penalty of the F136 substitution would be advantageous when the fungicide concentration was not high enough to saturate all CYP51 molecules. It would operate whether heteroallelism stemmed from gene duplication, heterokaryosis or merodiploidy.

Heteroallelism may have a similar advantage in *E. necator*, in which *Cyp51*‐heteroallelic isolates had higher azole resistance than F136 isolates, while the most insensitive isolates exhibited both heteroallelism and *Cyp51* overexpression (Rallos & Baudoin, 2016). In support of this hypothesis for the evolution of heteroallelic CYP51, the mean expression of Het+Het Bgt isolates was lower than the mean of F136+S509 isolates and the F136+T509 isolate, but the Het+Het genotype had higher mean ED_50_ values for both azoles, indicating that higher resistance was achieved in Het+Het isolates with lower gene expression. A rigorous test of this hypothesis should be conducted in an organism in which molecular genetic experiments are more tractable than *B. graminis*. Other means of mitigating detrimental effects of the [Y137F] substitution may operate in other fungi; in *Cyp51* from *Z. tritici* expressed in *S. cerevisiae*, Y137F is lethal to protein function but an additional S524T substitution largely restores CYP51 function (Cools et al., 2011).

In a second way that higher copy number could be adaptive for Bgt, a stoichiometric model may account for the moderate resistance to prothioconazole in some Y136+S509 isolates. The presence of more wild‐type *Cyp51* genes in Y136+S509 individuals is associated with higher expression of *Cyp51* (Figure 6b). Prothioconazole might thus be sequestered by some CYP51 molecules, while excess CYP51 could continue to demethylate lanosterol. This mechanism might not be effective against tebuconazole, which is more active against Bgt (compare ED_50_ values in Figure 1a,b). Again, this hypothesis should be tested in a more tractable fungus.

In research on *E. necator*, it was concluded that heteroallelism and increased copy number both correlated with heightened azole resistance (Jones et al., 2014; Rallos & Baudoin, 2016). In Bgt, however, heteroallelism and copy number are correlated but also have partially separate effects on azole resistance because F136+S509 and Het+S isolates in the United States had similar numbers of *Cyp51* genes, as did F136+S509 and Het+Het isolates in the UK. Rallos and Baudoin (2016) concluded that in *E. necator*, increased *Cyp51* copy number becomes advantageous only when the F136 allele has emerged in the fungal population but even without protein sequence alterations, greater copy number could contribute to azole resistance of Y136+S509 Bgt, at least to prothioconazole. Greater *Cyp51* copy number and expression have been implicated in azole resistance in fungi of medical importance including *Aspergillus fumigatus* (Khateb et al., 2023) and *C. albicans* (Selmecki et al., 2010).

There were three principal differences between the US and UK samples, with traits contributing to greater resistance being more common in the UK, including sequence mutations, heteroallelism and higher *Cyp51* numbers and expression. First, Y136+S509 isolates were abundant in the United States but were not detected in the smaller sample from the UK. Homoallelic F136+S509 isolates were present in both countries and one homoallelic F136+T509 isolate was collected in the UK. Second, in heteroallelic isolates, the mutant US alleles were Y136+S509/F136+S509 in Het+S509 isolates while in the UK, only the Het+Het genotype (Y136+S509/F136+T509) was found, not Het+S509. And third, both *Cyp51* copy number and expression were higher in the UK than in the United States. These features are all consistent with selection for higher resistance by greater use of azoles in the UK, where they have been used routinely as broad‐spectrum fungicides on wheat since the late 1970s.

In the United States, the frequency of the F136 residue in the field was higher in regions where the Bgt population had been shown previously to have lower levels of average azole sensitivity (Meyers et al., 2019). This adds empirical evidence to previous findings that the F136 substitution is a key mechanism for adaptation of powdery mildew fungi to DMI fungicides. Het+S509 isolates comprised a relatively small fraction of the Bgt population (9%), suggesting that the Het+S509 genotype may not have a large selective advantage over the more common F136+S509 under the typical US wheat fungicide regime, which generally involves only one or two applications per year. However, Het+S509 isolates were widely distributed throughout the Mid‐Atlantic and north‐east states and also in Mississippi and Kansas, west of the main wheat mildew zone. As prevailing wind patterns in the eastern United States are west to east (Cowger et al., 2016), the Het+S509 genotype may have emerged in diverse regional subpopulations by convergent evolution from separate mutation and gene duplication events. Winds in this region blow occasionally east to west, so it is also possible that Het+S509 emerged in an eastern state and then dispersed westwards, becoming recombined into diverse genetic backgrounds. In any event, the present study gives no reason to believe the T509 substitution has yet evolved in the United States.

The mean sporulation capacity of Het+S509 and Y136+S509 isolates was higher than F136+S509, suggesting that heteroallelism may partly restore fitness in azole‐resistant Bgt. There was wide variation, however, between isolates with each genotype, and the difference between allelic groups was not statistically significant. As in most studies of natural selection, it is difficult to measure small differences in fitness that may nonetheless be important over multiple generations. In addition, fitness costs may involve components other than conidium production, including features of both the asexual and sexual phases.

Taken together, these results illustrate that underlying the adaptation of Bgt to azole fungicides are multiple evolutionary processes. Four distinct routes to resistance could be discerned in the samples studied here: mutation in *Cyp51* sequences, heteroallelism, increased *Cyp51* copy number and expression in isolates with an azole resistance allele of *Cyp51*, and increased copy number in isolates with only a susceptible allele. We hypothesize that while the F136 and T509 substitutions are important steps for Bgt along the evolutionary pathway of azole resistance, they entail a fitness cost that has led to selection for compensatory traits, buffering any detrimental effect of CYP51 variants on fitness.

By providing a snapshot of the position of US and UK Bgt isolates along the evolutionary pathway of azole resistance, this study identifies key indicators by which resistance in powdery mildew from other cereal production areas and other crop species can be assessed. Surveillance of the genetic factors involved may be useful in encouraging US producers to take measures to reduce the risk of increased azole resistance such as that in the UK. Breeding for durable, quantitative mildew resistance has greatly reduced the significance of this wheat disease in the UK. Recommended measures in the United States include avoiding the planting of susceptible varieties in areas prone to powdery mildew, and rotating fungicide chemistries to reduce selection pressure on the pathogen population. This investigation presents the first strong evidence that the F136 substitution is associated with higher azole resistance in the United States, as previously shown for *B. graminis* in other regions and for other powdery mildew fungi.

Antimicrobial resistance is a current topic in microbiology, encompassing pathogens of crops, humans and livestock. It is widely recognized that the effectiveness of existing antimicrobials must be conserved while new modes of action are discovered. This requires careful monitoring of the advance of resistance in pathogen populations. DNA‐based surveys of resistance in crop‐pathogenic fungi have largely focussed on variation in inferred protein sequences, but our work indicates that this may be inadequate, and that informative surveys must also consider gene number, expression and possibly the presence of multiple alleles. This may apply in resistance to fungicides in general, not just triazoles.

## 4 EXPERIMENTAL PROCEDURES

### 4.1 Sample collection and isolate derivation

Three groups of Bgt isolates were studied. Firstly, the 363 US Bgt isolates were derived from samples collected from 27 commercial wheat fields in 15 states (Tables 1 and S7) in 2013 and 2014 as described in Cowger et al. (2018) and Meyers et al. (2019). Genetically pure isolates were obtained by two rounds of single‐spore subculturing and were maintained as described in Cowger et al. (2018). Results of tebuconazole and prothioconazole sensitivity screening were published previously (Meyers et al., 2019). From the 363 phenotyped US isolates, a subset of 30 isolates was selected for *Cyp51* expression and copy number assays representing the full range of geographic diversity and previously known variation in *Cyp51* (see Table S7 for details).

Secondly, the 46 UK isolates were all collected in Norfolk, UK. Thirty isolates were obtained from wheat in greenhouses at the JIC, Norwich, in 2014 and 2015, and 16 isolates from seedlings of the mildew‐susceptible wheat cv. Cerco placed in exposed locations at sites around Norfolk in 2015 (Table S1). Isolates were maintained on detached leaf segments of cv. Cerco on water agar (10 g L^−1^) containing benzimidazole (100 mg L^−1^). Genetically pure, single‐colony isolates, each derived from one conidium, were obtained by repeated subculturing (Brown & Wolfe, 1990). For the sake of comparison, 18 UK isolates were tested in experiments in the United States along with the US isolates, and similarly, 12 US isolates were tested in the UK along with the UK isolates.

Thirdly, four older isolates, JIW11, Fel09, 94202 and 96224, were used for reference (Table S1).

### 4.2 Determining Bgt sensitivity to tebuconazole and prothioconazole

In the United States, the same detached leaf assay previously used to test US isolates (Meyers et al., 2019) was used to measure tebuconazole and prothioconazole sensitivity of the 18 UK isolates, by spraying isolates to run‐off with a series of doses of each fungicide and recording symptoms on a 0–3 scale (Table S5). In the UK, isolates' responses to fungicides were tested by a precision spray method mimicking agricultural spraying and counting sporulating colonies formed 1 week later, as previously described (Brown & Evans, 1992) with minor modifications (Table S5). In both laboratories, ED_50_ values were calculated by nonlinear logistic regression of symptoms on log_10_(dose) (Table S6).

### 4.3 Genotyping of isolates and sequencing of *Cyp51*

DNA extraction was conducted in conventional ways in the two laboratories (File S1). The UK Bgt isolates were genotyped to identify probable clonal lineages using SSR markers Bgt‐5, Bgt‐8 and Bgt‐10 (Parks et al., 2011) and virulence on differential varieties (File S1) (Robinson et al., 2002).

For the 363 US isolates and the reference isolates 96224, JIW11 and Fel09, the entire *Cyp51* gene and its upstream promoter region were sequenced using the amplicon sequencing platform AmpSeq (Table S8; Yang et al., 2016). The heteroallelic *Cyp51* genotype was first observed as a double peak on Sanger sequencing chromatographs of a few isolates. The procedure used to confirm that the result was not due to contamination is described in File S1.

In the UK, primers were designed from the Bgt isolate JIW24 *Cyp51* gene sequence (GenBank ID AJ578751.1) to PCR‐amplify *Cyp51* and sequence the gene. Primer sequences, melting temperatures, and amplicon locations and sizes are shown in Table S9. An initial batch of sequencing revealed that several UK isolates appeared to have both Y136 and F136 in the CYP51 protein sequence, which was tested by cloning several copies of the complete *Cyp51* gene from each of these isolates (File S1).

### 4.4 Estimating *Cyp51* copy number

Estimates of *Cyp51* copy number were performed in both US and UK laboratories using the same ddPCR methodology (Hindson et al., 2011). In the United States, the 30 selected US isolates and the DMI‐sensitive reference isolate JIW11 were studied with three replicate extracts per isolate with each extract tested as one sample, with two technical replicates per gene per sample. In the UK, the technique was used to evaluate six US isolates, 16 UK isolates (of which eight were members of three glasshouse clonal lineages) and four reference isolates (JIW11, Fel09, 94202 and 96224). One DNA extract of each isolate was studied with one to three replicate samples per extract, with two or three technical replicates per gene per sample. Primer sequences, product sizes and locations within genes for both genes are shown in Table S10, while details on analysis of variation in *Cyp51* copy number between Bgt genotypes and countries performed by linear mixed modelling are in Table S3.

### 4.5 Quantifying *Cyp51* gene expression using RT‐qPCR and RNA‐seq

In both laboratories, *Cyp51* expression in the absence of an azole fungicide was assessed by RT‐qPCR for the isolates listed in Table S1. Details are in Table S11. The statistical method of using *C*_q_ and primer efficiency values to estimate *Cyp51* expression relative to that of the reference genes is described in Table S4. In the UK only, *Cyp51* expression was also assessed by RNA‐seq in 31 isolates, including the four reference isolates (Table S1). Details on RNA‐seq methodology are in Table S11.

### 4.6 Relationship of triazole resistance to other variables

Principal component analysis was done with data on eight traits: log_10_ED_50_ values of tebuconazole and prothioconazole as predicted means across laboratories, *Cyp51* copy number, *Cyp51* expression quantified by RT‐qPCR as described above, the amino acid at residues 136 and 509 of CYP51, and homoallelism or heteroallelism at the same two residues. To enable sequence data to be included in the analysis, variants were coded as numerical variables with the allele associated with increasing triazole resistance given a value of 1 (F136, T509, heteroallelism at both residues) and the wild‐type allele coded as 0 (Y136, S509, homoallelism). Data on the 49 isolates that were scored for all relevant traits were included. The analysis was done with the PCP directive of GenStat 22nd edition.

## CONFLICT OF INTEREST STATEMENT

The authors have no conflicts of interest.

## Supporting information


**Figure S1.** Sporulation of US isolates with varying *Cyp51* genotypes.
**File S1.** DNA extraction, mutations not linked to phenotype, heteroallelism tests.

**Figure S1.** Sporulation of US isolates with varying *Cyp51* genotypes.
**File S1.** DNA extraction, mutations not linked to phenotype, heteroallelism tests.

**Table S1.** Isolates of *Blumeria graminis* f. sp. *tritici* used in this study.

**Table S2.** Apparently clonal lineages of Bgt in the UK.

**Table S3.** Statistical analysis of data on *Cyp51* copy number in *Blumeria graminis* f. sp. *tritici*.

**Table S4.** Statistical analysis of data on *Cyp51* gene expression in *Blumeria graminis* f. sp. *tritici*.

**Table S5.** Traits of *Blumeria graminis* f. sp. *tritici* isolates.

**Table S6.** Statistical analysis of median effective doses (ED_50_) of tebuconazole and prothioconazole.

**Table S7.** US *Blumeria graminis* f. sp. *tritici* isolates genotyped from each of 27 fields.

**Table S8.** Primers for amplification and sequencing of *Cyp51* in the United States.

**Table S9.** Primers for amplification and sequencing of *Cyp51* in the UK.

**Table S10.** Primers and product sizes for estimating *Cyp51* copy number.

**Table S11.** Primers and other details for reverse transcription‐quantitative PCR estimation of *Cyp51* expression.

**Table S12.** Statistical analysis of US isolate sporulation.

## Data Availability

*Cyp51* sequences are available from NCBI (in GenBank, accession numbers are OR763808–OR763813 for USDA sequences, and OR753405–OR753408 for JIC sequences). The raw transcriptomic sequence data have been deposited in the European Nucleotide Archive (project accession number PRJEB68334). Pathology data are available from the authors on request.
