# Supplementary material for: Multiple routes to fungicide resistance: Interaction of Cyp51 gene sequences, copy number and expression
Source: Mol Plant Pathol. 2024 Sep 20;25(9):e13498. doi: 10.1111/mpp.13498 (PMC11415427; doi:10.1111/mpp.13498)
Supplement: Supplementary file 1 — Figure S1. Sporulation of US isolates with varying Cyp51 genotypes. File S1. DNA extraction, mutations not linked to phenotype, heteroallelism tests. [file MPP-25-e13498-s003.docx]

# Multiple routes to fungicide resistance: interaction of *Cyp51* gene sequences, copy number and expression

Corinne J. Arnold, Emily A. Meyers, Rebecca Whetten, Laetitia Chartrain, Jitender Cheema,

James K.M. Brown, and Christina Cowger

**SUPPORTING INFORMATION**

**TABLE Page**

**Table S1** Isolates of *Blumeria graminis* f. sp. *tritici* (*Bgt*) used in this study…………………2

**Table S2** Apparently clonal lineages of *Bgt* in the UK………………………………….….. 5

**Table S3** Statistical analysis of data on *Cyp51* copy number in *Bgt*…………………………. 6

**Table S4** Statistical analysis of data on *Cyp51* gene expression in *Bgt*…………………….... 8

**Table S5** Traits of *Bgt* isolates ……………………………………………………………….10

**Table S6** Statistical analysis of median effective doses (ED50) of tebuconazole and prothioconazole …………………………………………………………………………….12

**Table S7** US *Bgt* isolates genotyped from each of 27 fields ………………………………...14

**Table S8** Primers for amplification and sequencing of *Cyp51* in the US………………….... 16

**Table S9** Primers for amplification and sequencing of *Cyp51* in the UK……………………17

**Table S10** Primers and product sizes for estimating *Cyp51* copy number…………………...18

**Table S11** Primers and other details for qRT-PCR estimation of *Cyp51* expression ………..19

**Table S12** Statistical analysis of US isolate sporulation …………………………………… 21

**File S1** DNA extraction, mutations not linked to phenotype, and heteroallelism tests……… 22

**Figure S1** Sporulation of US isolates with varying Cyp51 genotypes……………………… .25

**References** …………………………………………………………………………………... 26
