# Supplementary material for: Multiple routes to fungicide resistance: Interaction of Cyp51 gene sequences, copy number and expression
Source: Mol Plant Pathol. 2024 Sep 20;25(9):e13498. doi: 10.1111/mpp.13498 (PMC11415427; doi:10.1111/mpp.13498)
Supplement: Supplementary file 2 — Figure S1. Sporulation of US isolates with varying Cyp51 genotypes. File S1. DNA extraction, mutations not linked to phenotype, heteroallelism tests. [file MPP-25-e13498-s009.zip › Figure S1.docx]

Het

(n = 32)

F136

(n = 91)

Y136

(n = 221)

**FIGURE S1**

Sporulation (conidia per colony) as a fitness trait measured on 351 US *Bgt* isolates. *Cyp51* genotypes: Y136 = wild type, F136 = mutant (Y136F), Het = Y136+S509 and F136+S509. Blue dots: individual isolates. Orange dots and bars: mean and standard errors of each group.
