# Supplementary material for: Multiple routes to fungicide resistance: Interaction of Cyp51 gene sequences, copy number and expression
Source: Mol Plant Pathol. 2024 Sep 20;25(9):e13498. doi: 10.1111/mpp.13498 (PMC11415427; doi:10.1111/mpp.13498)
Supplement: Supplementary file 2 — Figure S1. Sporulation of US isolates with varying Cyp51 genotypes. File S1. DNA extraction, mutations not linked to phenotype, heteroallelism tests. [file MPP-25-e13498-s009.zip › File S1.docx]

**File S1.** DNA extraction, mutations not linked to phenotype, and heteroallelism tests

**DNA extraction**

In the USA, conidial DNA was extracted from the 363 US isolates and the control isolates JIW11, Fel09, and 96224 with an E.Z.N.A.® Plant DNA extraction kit (Omega Bio-Tek, Norcross, GA). Conidia were collected into 2 mL screwcap tubes by dipping pustule-covered leaf segments in 100% ethanol. Conidial samples were then centrifuged for 20 min and ethanol was decanted from the tubes. The conidial samples were frozen at −80°C, lyophilized to remove all remaining ethanol, and stored at −80°C until DNA extraction. Three replicate tubes containing approximately equal volumes of conidia were collected per isolate. Samples were ground twice about 30 s each time with five nickel-plated beads per tube, keeping them in liquid nitrogen between grinding sessions, and agitated with a Vortex Genie attachment. DNA was quantified using a Qubit 2.0 fluorometer (ThermoFisher).

In the UK, two methods of DNA extraction were used. For analysis of microsatellite markers, mating type alleles (K. McNally, personal communication) and *Cyp51* sequences, DNA was extracted from three detached leaves infected with a mildew isolate, 12-14 days after infection. Leaves were wrapped in tinfoil, frozen in liquid N_2_ and ground to a fine powder using a pestle, mortar and a small amount of sand. For ddPCR, conidia were harvested from each isolate, then ground using a pestle, mortar, a small amount of sand, and liquid nitrogen. In both cases, DNA was extracted using a QIAGEN DNeasy Mini Plant Extraction Kit according to the manufacturer’s protocol. DNA from infected leaves was quantified using a NanoDrop2000 spectrophotometer, although this included DNA from the leaf as well as the fungus. In extracts from conidia, DNA was quantified using a Qubit™ 3.0 fluorometer (Invitrogen, UK) and the Qubit™ dsDNA HS Assay kit (Invitrogen, UK).

**Mutations not linked to phenotype**

Besides F136, only one other CYP51 substitution relative to reference isolate 96224, K175N, was detected in the USA. This variant was not observed in the present set of UK isolates but a previous UK sample was polymorphic for K175 and N175 (Wyand and Brown 2005), with both azole-resistant and -susceptible isolates having both variants of the residue. Thus, while a relationship of residue 175 to azole sensitivity cannot be entirely excluded, it appears unlikely. In addition, US isolates had a SNP in *Cyp51* intron 2 that UK isolates did not have.

Amplicon 6, containing codon 509, was sequenced in all UK isolates which had F136 but lacked Y136. One of these had T509 (F+T allele) while the remainder had S509 (F+S). No non-synonymous variation at codons other than 136 or 509 was detected in *Cyp51* gene sequences from UK isolates. In particular, all isolates for which the complete *Cyp51* gene was sequenced were wild-type at codons 171, 301 and 327, where variation has been reported in *Bgh* from Western Australia (Tucker et al. 2019).

Among the complete *Cyp51* sequences from UK isolates, the majority had L12 as in *Bgt* sequences reported previously (Wyand and Brown 2005) but two had an L12C [L12C] substitution in the N-terminal amphipathic alpha-helix. Relative to all previously sequenced isolates including Fel09 and JIW11 (Wyand and Brown 2005), all the complete sequences from UK isolates as well as 96224 had H398N [H404N], G430S [G436S], N497E [N512E], S498G [S513E] and T516I [T531I] substitutions. These were all in external loops of the CYP51 protein and were not in substrate recognition sites (Chartrain and Brown 2023).

**Heteroallelism tests**

In the UK, the complete *Cyp51* gene was amplified using primers Blumeria_CYP51_Amp1.1 and Bg_CYP51Amp6.2R and purified. Thermocycling conditions were 98 °C for 30 s, followed by 33 cycles of 98 °C for 10 s, 57 °C for 20 s, and 72 °C for 50 s, with a final extension at 72 °C for 2 min. For cloning, A-overhangs were added to PCR products by adding 5 µL purified PCR product to 1 µL 10x standard Taq reaction buffer, 2 µL dATP (final concentration 2 mM), 1 µL Taq DNA polymerase (New England BioLabs Inc., UK; total of 10 units), and 1 µL water, incubated at 70 °C for 30 min. PCR products were ligated into the pGEM®-T easy vector (Promega UK) in 11 µL reactions containing 1x rapid ligation buffer, 25 ng pGEM®-T easy vector, 1.5 U T4 DNA ligase, 2 µL unpurified A-tailing reaction, and made up to 11 µL with water. Reactions were incubated at room temperature for 1 hr, then overnight at 4 °C. Aliquots of 10 μL of each ligation reaction were transformed into 50 µL Subcloning Efficiency™ DH5α cells (Invitrogen, UK) according to the manufacturer’s protocol. LB agar plates supplemented with ampicillin, Xgal, and IPTG were made from InvivoGen *E. coli* Fast Media sachets, 125 µL cells were spread on each plate, and plates were incubated overnight at 37 °C. Individual white colonies were picked with a sterile toothpick and incubated in 5 mL LB with 200 µg/mL carbenicillin shaken at 220 rpm overnight at 37 °C. Plasmid was isolated from individual liquid cultures using a QIAGEN QIAprep Spin Miniprep Kit according to the manufacturer’s protocol and quantified using a NanoDrop2000 spectrophotometer. Samples were sequenced by Eurofins Genomics with primers Blumeria_CYP51_Amp1.1, -2.2, -3.1, Bg_CYP51Amp6.1R, and Bg_CYP51Amp6.2R. Four clones from every 2014 glasshouse isolate, nine ADW1501 clones, and seven EOW1501 clones were sequenced in this way.

Amplicon 2, containing codon 136 of *Cyp51*, was sequenced in all UK isolates from 2014 and 2015, which had either F136 (TTT) or both Y and F (TAT and TTT) at residue 136; none had Y136 only. To test whether isolates with both alleles were genetically heteroallelic or mixtures of different genotypes, UK isolate CAW15S6323 with both Y136 and F136 was subcultured as seven single-colony isolates and three US Het+S isolates as five single-spore isolates each. The complete *Cyp51* gene was sequenced in these 22 single-colony isolates, revealing that both Y136 and F136 were present in all these subcultured isolates.

All *Cyp51* gene sequences from the CAW15S6323 single-colony isolates which had F136 also had T509 rather than S509. By contrast, all Y136 gene sequences from CAW15S6323 had S509, as did all US isolates, whether they had Y136 or F136. A further four Y/F136 glasshouse isolates from the UK were subcultured to obtain four single-colony isolates each, while nine and seven single-colony isolates were obtained from two UK Y/F136 field isolates. In the complete *Cyp51* gene sequence from these single colony isolates, the ratios of the Y136:F136 alleles were 2:2 in subcultures of each glasshouse isolate, and 5:4 and 5:2 in the two field isolates. F136 was invariably associated with T509 in these sequences and Y136 with S509. This indicates that Y136/F136 Het isolates from the UK were also heteroallelic for S509/T509 (Het+Het), the alleles present encoding Y136+S509 and F136+T509.

In the US, the heteroallelic (Het) genotype possessing both TAT and TTT codons was first observed as a double peak on Sanger sequencing chromatographs of a few isolates. These results were confirmed not to be due to isolate cross-contamination by growing five single-spored subsamples from each of three isolates and sequencing each subsample. All subsamples showed the same double peak on the chromatograph, indicating both Y136 and F136 were present in these isolates. The Het genotype was further confirmed during amplicon sequencing.

**References**

Chartrain, L., and Brown, J. K. M. 2023. Molecular evolution and mechanisms of fungicide resistance in plant pathogenic fungi. Pages 400 in: Understanding and minimising fungicide resistance. Eds: Lopez-Ruiz, F. Burleigh Dodds Science Publishing Limited, Cambridge, United Kingdom. doi:<http://dx.doi.org/10.19103/AS.2022.0116.02>.

Tucker, M. A., Lopez-Ruiz, F., Cools, H. J., Mullins, J. G., Jayasena, K., and Oliver, R. P. 2019. Analysis of mutations in West Australian populations of *Blumeria graminis* f. sp. *hordei CYP51* conferring resistance to DMI fungicides. Pest Manag. Sci. 76:1265-1272.

Wyand, R. A., and Brown, J. K. 2005. Sequence variation in the CYP51 gene of *Blumeria graminis* associated with resistance to sterol demethylase inhibiting fungicides. Fungal Genet. Biol. 42:726-35.
