# Supplementary material for: Multiple routes to fungicide resistance: Interaction of Cyp51 gene sequences, copy number and expression
Source: Mol Plant Pathol. 2024 Sep 20;25(9):e13498. doi: 10.1111/mpp.13498 (PMC11415427; doi:10.1111/mpp.13498)
Supplement: Supplementary file 3 — Table S1. Isolates of Blumeria graminis f. sp. tritici used in this study. [file MPP-25-e13498-s002.pdf]

Table S1 Isolates of *Blumeria graminis* f.sp. *tritici* used in studies of genetic variation in *Cyp51* (*Erg11*) in relation to resistance to azole fungicides. (a) US isolates for which gene expression, gene number or both were estimated are included, together with (b) all UK isolates studied and (c) four reference isolates.

Isolate	Residue 136+509	Source	Clonal lineage	ED50 tebuconazole (mean of labs) [1]	ED50 prothioconazole (mean of labs) [1]	Expression of *Cyp51* / control genes (qRT-PCR)	Cyp51 transcription (FPKM)	Estimated number of Cyp51 genes	ED50 tebuconazole, mg/L (USDA)	ED50 prothioconazole, mg/L (USDA)	ED50 Folicur (TBC) mL/ha (JIC)	ED50 Proline (PTC) mL/ha (JIC)
(a) Isolates from the USA, 2013-14												
ALA2-E-1-1	Y+S	Alabama		2.64	19.5	0.475		2.1	1.37	23.4		
ARF-A-1-5	Y+S	Arkansa		3.59	60.6	0.168		0.90	1.86	72.5		
FLG-B-1-3	Y+S	Florida		0.797	5.70	0.137		0.72	0.413	6.81		
GAS-B-1-2	Y+S	Georgia		3.49	36.3	0.137		0.98	1.81	43.4		
KSM C.2.4	Y+S	Kansas		1.30	22.9		83.3		0.225	12.7	10.8	37.5
KSM-B-1-5	Y+S	Kansas		3.40	75.3	0.160		0.70	1.76	90.1		
KSM-C-2-5	Y+S	Kansas		1.43	62.6	0.0684	84.6	0.80	0.294	86.3	9.97	41.3
KSM-C-3-4	Y+S	Kansas		1.30	35.4	0.0593	51.6	0.58	0.222	17.7	10.9	64.3
MIR(14)-C-2-1	Y+S	Michigan		0.517	46.7	0.279		3.9	0.268	55.8		
MOB(14)-A-2	Y+S	Missouri		0.492	9.52	0.177		1.1	0.255	11.4		
MOB(14)-B-1	Y+S	Missouri		0.664	6.59		74.2		0.345	7.88		
MOB(14)D2	Y+S	Missouri		2.15	34.4		81.3		0.534	19.7	12.5	54.5
MSG-C-3-4	Y+S	Mississippi		8.11	123	0.408		4.0	4.21	147		
NCF-D-1-1	Y+S	N.Carolina		1.48	34.6	0.47		3.6	0.77	41.4		
NEL-6	Y+S	Nebraska		4.33	24.4	0.226	74.0	0.91	2.36	13.8	11.4	39.2
NYB(14)-C-3-1	Y+S	New York		1.91	21.6	0.368		1.9	0.989	25.8		
OHJ-C-3-3	Y+S	Ohio		0.298	9.80	0.101		0.73	0.154	11.7		
OHW-D-3-5	Y+S	Ohio		14.4	230	0.37		4.0	7.44	275		
OKH-A-2-1	Y+S	Oklahoma		2.98	72.6		66.2		1.54	86.7		
OKS(14)-A-2-3	Y+S	Oklahoma		0.900	21.5	0.125	71.2	1.5	0.118	8.13	9.9	51.9
OKS(14)B.3.5	Y+S	Oklahoma		1.12	25.6		66.0		0.187	12.4	9.56	48.1
OKS-A-2-2	Y+S	Oklahoma		1.34	35.0	0.163		0.93	0.697	41.9		
OKS-B-1-4	Y+S	Oklahoma		1.86	9.93		91.0		0.964	11.9		
PAF(14)-B-1-4	Y+S	Pennsylvania		2.90	269	0.169		3.6	1.51	322		
GAP-B-2-2	F+S	Georgia		21.7	67.5	0.202	394	1.6	11.3	80.8		
GAT-A-3-4	F+S	Georgia		10.8	195	0.232	305	3.0	4.02		41.9	148
GAT-B-2-4	F+S	Georgia		8.49	87.4	0.404		1.8	4.4	105		
GAT-D-3-3	F+S	Georgia		18.1	56.4	0.466		2.8	9.39	67.5		
MIR(14)-D-3-3	F+S	Michigan		0.702	6.52	0.163		0.72	0.36	7.80		
NCM-A-1-1	F+S	N.Carolina		1.53	90.9	0.193		0.90	0.795	109		
NCM-B-3-4	F+S	N.Carolina		31.3	173	0.373		1.8	16.2	207		
NYA-E-3-3	F+S	New York		5.44	21.4	0.185		0.74	2.82	25.6		
NYG-B-1-5	F+S	New York		5.18	159	0.244		2.0	2.69	191		
NYG-D-1-1	F+S	New York		9.84	32.0	0.397		2.0	5.1	38.3		
KSM-B-3-1	Y+S/F+S	Kansas		8.16	72.2	0.389		2.8	4.23	86.3		

Table S1, continued (page 2) Isolates of *Blumeria graminis* f.sp. *tritici* used in studies of genetic variation in *Cyp51* (*Erg11*) in relation to resistance to azole fungicides. (a) US isolates for which gene expression, gene number or both were estimated are included, together with (b) all UK isolates studied and (c) four reference isolates.

Isolate	Residue 136+509	Source	Clonal lineage	ED50 tebuconazole (mean of labs) [1]	ED50 prothioconazole (mean of labs) [1]	Expression of *Cyp51* / control genes (qRT-PCR)	Cyp51 transcription (FPKM)	Estimated number of Cyp51 genes	ED50 tebuconazole, mg/L (USDA)	ED50 prothioconazole, mg/L (USDA)	ED50 Folicur (TBC) mL/ha (JIC)	ED50 Proline (PTC) mL/ha (JIC)
KSM-D-1-5	Y+S/F+S	Kansas		19.0	34.5	0.427		2.9	9.86	41.2		
NCM-E-1-2	Y+S/F+S	N.Carolina		19.3	199	0.387		2.5	10	237		
VAS-B-1-3	Y+S/F+S	Virginia		2.65	82.2	0.319		2.6	1.37	98.3		
VAS-B-3-2	Y+S/F+S	Virginia		3.12	60.4	0.356		2.4	1.62	72.2		
VAS-D-2-1	Y+S/F+S	Virginia		2.69	43.0	0.426		2.5	1.39	51.4		
(b) Isolates from the UK, 2014-15												
CAW lineage 1	F+S	JIC glasshouses		49.0	465.0	0.816	2360	3.3	45.8	854	75.3	230
CAW14S6101	*F+S*	*JIC glasshouses*	*1*			*0.565*		*3.9*			*127*	*278*
CAW14S6102	*F+S*	*JIC glasshouses*	*1*									*138*
CAW14S6104	*F+S*	*JIC glasshouses*	*1*									*466*
CAW14S6302	*F+S*	*JIC glasshouses*	*1*									*371*
CAW14S6303	*F+S*	*JIC glasshouses*	*1*			*0.436*	*2070*	*2.5*	*34.4*	*921*	*104*	*249*
CAW14S6304	*F+S*	*JIC glasshouses*	*1*			*0.393*					*51.8*	*151*
CAW14S6305	*F+S*	*JIC glasshouses*	*1*									*194*
CAW14S6306	*F+S*	*JIC glasshouses*	*1*									*446*
CAW14S6307	*F+S*	*JIC glasshouses*	*1*			*0.835*					*58.5*	*257*
CAW14S6308	*F+S*	*JIC glasshouses*	*1*			*0.892*					*94.2*	*132*
CAW14S6310	*F+S*	*JIC glasshouses*	*1*									*360*
CAW14S6311	*F+S*	*JIC glasshouses*	*1*			*0.979*					*125*	*280*
CAW14S6316	*F+S*	*JIC glasshouses*	*1*									*316*
CAW14S6318	*F+S*	*JIC glasshouses*	*1*			*0.686*			*61.2*	*792*	*88.2*	*428*
CAW15S6332	*F+S*	*JIC glasshouses*	*1*			*0.413*	*2700*	*3.8*			*60*	*160*
CAW lineage 2	F+S	JIC glasshouses		31.6	316	0.584	2360	2.5	22.1	384	64.8	236
CAW14S6301	*F+S*	*JIC glasshouses*	*2*			*0.589*		*1.7*	*22.1*	*384*	*63.5*	*370*
CAW14S6313	*F+S*	*JIC glasshouses*	*2*			*0.573*	*2880*				*69.5*	*236*
CAW15S6330	*F+S*	*JIC glasshouses*	*2*			*1.70*	*1930*	*3.7*			*75.2*	*205*
CAW lineage 3	Y+S/F+T	JIC glasshouses		81.1	465	0.483	337	2.7	70.6	624	134	315
CAW14S6309	*Y+S/F+T*	*JIC glasshouses*	*3*			*0.374*	*238*	*2.3*	*70.6*	*624*	*201*	*406*
CAW14S6317	*Y+S/F+T*	*JIC glasshouses*	*3*			*0.564*	*476*	*3.0*			*137*	*392*
CAW14S6317	*Y+S/F+T*	*JIC glasshouses*	*3*			*1.10*		*2.9*			*138*	*119*
CAW14S6317	*Y+S/F+T*	*JIC glasshouses*	*3*			*0.361*					*139*	*292*
CAW lineage 5	F+S	JIC glasshouses		21.3	168		1890				59.1	128
CAW15S6334	*F+S*	*JIC glasshouses*	*5*				*1530*				*68.8*	*174*
CAW15S6343	*F+S*	*JIC glasshouses*	*5*				*2320*				*49.7*	*116*
CAW15S6320	*F+S*	*JIC glasshouses*		*21.7*	*244*		*1400*				*60.1*	*186*

Table S1, continued (page 3) Isolates of *Blumeria graminis* f.sp. *tritici* used in studies of genetic variation in *Cyp51* (*Erg11*) in relation to resistance to azole fungicides. (a) US isolates for which gene expression, gene number or both were estimated are included, together with (b) all UK isolates studied and (c) four reference isolates.

Isolate	Residue 136+509	Source	Clonal lineage	ED50 tebuconazole (mean of labs)[1]	ED50 prothioconazole (mean of labs)[1]	Expression of *Cyp51* / control genes (qRT-PCR)	Cyp51 transcription (FPKM)	Estimated number of Cyp51 genes	ED50 tebuconazole, mg/L (USDA)	ED50 prothioconazole, mg/L (USDA)	ED50 Folicur (TBC) mL/ha (JIC)	ED50 Proline (PTC) mL/ha (JIC)
RBPT22	F+S	JIC glasshouses		22.8	256	0.742	1430				63.3	195
CAW14S6103	F+T	JIC glasshouses		83.9	269	0.783	690	2.3			233	205
CAW15S6323	Y+S/F+T	JIC glasshouses		60.9	572	1.02			52.8	756	101	394
CAW15S6340	Y+S/F+T	JIC glasshouses		44.7	281	0.395					124	214
CAW15S6341	Y+S/F+T	JIC glasshouses		124	651	1.13		5.3			345	496
ADW1503	F+S	Norfolk		39.5	283	0.889			40.3	638	55.6	114
AEW1503	F+S	Norfolk		53.1	405	0.736			50.2	750	80.7	199
ASW1505	F+S	Norfolk		54.9	328	2.28			50.6	679	85.6	145
ASW1506	F+S	Norfolk		36.6	418	0.980		3.8			101	318
CMW1503	F+S	Norfolk		32.6	311	0.711			14	465	109	189
EOW1502	F+S	Norfolk		41.5	422	0.886		3.9	25.6	600	96.7	270
JBW1502	F+S	Norfolk		48.8	575						135	438
TKW1506	F+S	Norfolk		32.5	295	0.610			23.4	465	65.2	170
ADW1501	Y+S/F+T	Norfolk		82.6	500	0.231			37.7	893	260	255
AEW1504	Y+S/F+T	Norfolk		50.0	290	0.277		2.8	46.3	483	77.8	159
ASW1502	Y+S/F+T	Norfolk		97.1	272	0.339	1030	3.9	56.5	310	240	217
ASW1504	Y+S/F+T	Norfolk		23.7	275		573				65.7	209
CMW1502	Y+S/F+T	Norfolk		98.0	350	0.429	864	2.7	78.1	352	177	316
EOW1501	Y+S/F+T	Norfolk		115	689	0.604	1180	2.2	93.3	755	202	573
JBW1504	Y+S/F+T	Norfolk		129	404	0.721			93	708	257	210
TKW1504	Y+S/F+T	Norfolk		163	739	0.422			121	920	313	541
(c) Reference isolates [2]												
94202	Y+S	Switzerland, 1994		10.9	160	0.467	364	1.9			30.3	122
96224	Y+S/F+T	Switzerland, 1996		10.5	169	0.155	289	1.6			29.1	129
Fel09	F+S	Germany, c.1998		18.7	244	0.528	1200	2.8	13.9	463	36.0	117
JIW11	Y+S	UK, 1985		12.7	197	0.215	293	2.1	7.10	500	32.6	70.5

[1] Arbitrary units (see Table S6).

[2] Two reference isolates, the DMI-sensitive JIW11 and the partially resistant Fel09, were used in previous work on DMI resistance in *Bgt* (Wyand & Brown, 2005). Reference isolates 94202 and 96224, used for genetic research (Wicker et al., 2013), were kindly provided by Prof Dr B. Keller, University of Zürich.
