# Supplementary material for: Multiple routes to fungicide resistance: Interaction of Cyp51 gene sequences, copy number and expression
Source: Mol Plant Pathol. 2024 Sep 20;25(9):e13498. doi: 10.1111/mpp.13498 (PMC11415427; doi:10.1111/mpp.13498)
Supplement: Supplementary file 4 — Table S2. Apparently clonal lineages of Bgt in the UK. [file MPP-25-e13498-s007.docx]

**Table S2.** Apparently clonal lineages of *Blumeria graminis* f.sp. *tritici* isolated from glasshouses at the John Innes Centre, Norfolk, England in 2014 and 2015, characterised by single-sequence repeats (SSR), mating type, amino-acid 136 of CYP51 / ERG11 and virulence to *Pm3d* in cultivars Axona and Broom^a^

| Lineage | Number of isolates | | SSR marker size (bp) | | | Mating type | CYP51 residues | | Virulence to *Pm3d* |
| --- | --- | --- | --- | --- | --- | --- | --- | --- | --- |
|  | 2014 | 2015 | Bgt-5 | Bgt-8 | Bgt-10 |  | 136 | 509 |  |
| 1 | 14 | 1 | 250 | 530 | 507 | 2 | F | S | Virulent |
| 2 | 2 | 1 | 250 | 535 | 507 | 2 | F | S | Virulent |
| 3 | 4 | 0 | 281 | 448 | 594 | 1 | Y+F | S+T | Avirulent |
| 5 | 0 | 2 | 281 | 390 | 521 | 1 | F | S | Virulent |

^a^Variation in UK isolates was characterised by SSR variation and mating type. For SSR markers, the forward primers were M13-labelled in combination with the fluorophore HEX (Bgt-5, Bgt-10) or FAM (Bgt-8). PCR amplification was done with 1x HotStarTaq Master Mix (Qiagen Ltd, UK), 0.75 μM fluorolabelled adapter, 0.75 μM reverse primer, 0.05 μM forward primer with M13 tail, and 10 ng DNA, made up to 6.2 μL with DNAse-free water. Thermocycling conditions for Bgt-5 and Bgt-8 were 95 °C for 15 min followed by 40 cycles of 95, 58 and 72 °C for 1 min each, then a final extension step at 72 °C for 10 min. Bgt-10 required touchdown PCR with 95 °C for 15 min, followed by 10 cycles of 95 °C for 1 min, 65 °C to 56 °C for 1 min decreasing by 1 °C each cycle, and 72 °C for 1 min; this was followed by 24 cycles of 95, 55 and 72 °C for 1 min each, then a final extension step as above. For virulence analysis, the differential varieties were those used by Robinson et al. (2002) except that Hope (*Pm5*) was omitted and Shamrock (unknown mildew resistance gene) was included.

All isolates had an alanine codon at residue 143 of cytochrome b, indicating that they were resistant to quinone-outside inhibitor (QoI) fungicides, including strobilurin analogues (Robinson et al. 2002). Lineages 1 and 2, obtained in both 2014 and 2015, differed only by 5 bp in SSR marker Bgt‑8 and both had the F+S allele of *Cyp51*. They differed in all three SSR markers and mating type from Lineages 3 and 5, which differed from each other in three ways: by large amounts in SSR markers Bgt-8 and Bgt-10 and in their *Cyp51* allele because Lineage 3 was Het+Het whereas Lineage 5 was F+S. Lineage 3 was only collected in 2014 and Lineage 5 only in 2015. Lineage 3 was avirulent to *Pm3d* (infection type [IT] 0) while Lineage 1, 2 and 5 were virulent (IT 4). All four of these lineages were virulent to cultivars Galahad (*Pm2*), Aquila (*Pm5*), Holger (*Pm6*), Ambassador (*Pm8*) and Maris Dove and avirulent to Anfield (*Pm1*), Chul (*Pm3b*), Normandie (*Pm9*+*1*+*2*), Sicco and Wembley. All remaining glasshouse isolates and all isolates from the natural air spora differed from each other and from the four identified clonal lineages in one or more of these characteristics, and are thus each assumed to represent a different clone.

In statistical analyses, the Lineage term refers to unique clonal lineages, with the four UK glasshouse lineages, all unique UK isolates and all US isolates considered to be different Lineages. The Isolate term thus refers to variation between isolates within the four UK clonal lineages.

**REFERENCE**

Robinson, H. L., Ridout, C. J., Sierotzki, H., Gisi, U., and Brown, J. K. M. 2002. Isogamous, hermaphroditic inheritance of mitochondrion-encoded resistance to Qo inhibitor fungicides in *Blumeria graminis* f. sp. *tritici*. Fungal Genet. Biol. 36:98-106.
