# Supplementary material for: Multiple routes to fungicide resistance: Interaction of Cyp51 gene sequences, copy number and expression
Source: Mol Plant Pathol. 2024 Sep 20;25(9):e13498. doi: 10.1111/mpp.13498 (PMC11415427; doi:10.1111/mpp.13498)
Supplement: Supplementary file 5 — Table S3. Statistical analysis of data on Cyp51 copy number in Blumeria graminis f. sp. tritici. [file MPP-25-e13498-s005.docx]

**Table S3.** Statistical analysis of data on *Cyp51* copy number in *Bgt* obtained by droplet digital PCR (ddPCR).

| a) Variance components of random effects ^a^ | | |
| --- | --- | --- |
| **Random term** | **Component** | **s.e.** |
| Sample | 0.04197 | 0.00988 |
| Sample:Gene | 0.01992 | 0.00486 |
| Sample:TechRep | 0.00055 | 0.00151 |
| Sample:Gene:TechRep | 0.0166 | 0.00202 |

| b) Analysis of variance of fixed effects | | | |
| --- | --- | --- | --- |
| **Fixed term** | **d.f.** | **Wald statistic / d.f.** | **P (chi-sq)** |
| Gene | 1 | 880.05 | <0.001 |
| Lineage | 48 | 12.78 | <0.001 |
| Gene.Lineage | 48 | 16.48 | <0.001 |
| Clone.Isolate | 5 | 1.38 | 0.2 |
| Gene.Lineage.Isolate | 5 | 2.21 | 0.05 |

^a^ Abbreviations: s.e., standard error; d.f., degrees of freedom; P; chi-squared test probability.

**Notes**

The variable analysed was log_e_ [log_e_(Accepted­*_itg_*) – log_e_(Negative*_itg_*)] for gene *g* in technical replicate *t* of isolate *i*. ‘Gene’ is either *Cyp51* or the β-tubulin gene *Tub2*.

The number of amplified fragments in a droplet follows a Poisson distribution (Bio-Rad Laboratories);

<https://www.bio-rad.com/webroot/web/pdf/lsr/literature/Bulletin_6407.pdf>). If *F* is the number of fragments in a droplet, the fraction of droplets with no fragment (Negative/Accepted) is exp(−*F*). log_e_ (Accepted / Negative) is therefore an estimate of *F*. The number of genes of *Cyp51* relative to *Tub2* is therefore

*F_Cyp51_* / *F_Tub2_* = log_e_ (Accepted*_Cyp51_* / Negative*_Cyp51_*) / log_e_ (Accepted*_Tub2_* / Negative*_Tub2_*)

To avoid the usual problem of heteroscedastic residuals when analysing a variable which is a ratio, this quotient was log-transformed so the difference:

log_e_ [log_e_ (Accepted*_Cyp51_* / Negative*_Cyp51_*)] – log_e_ [log_e_ (Accepted*_Tub2_* / Negative*_Tub2_*)]

is the logarithm of the number of *Cyp51* genes in the isolate, given that *B. graminis* has one *Tub2* gene (Sherwood and Somerville 1990).

This variable was used in fitting the following linear mixed model:

Fixed effects: (Lineage/ Isolate) * Gene

Random effects: Sample / (Gene * TechRep)

Genetically distinct isolates are treated as different Lineages, each containing one isolate. The presumed clonal lineages of UK glasshouse isolates were each considered to belong to a distinct clone containing two or more isolates. The Isolate fixed effect therefore relates to variation between isolates within UK glasshouse clones 1, 2, 3 and 5. The Gene term is *Cyp51* or *Tub2*.

Sample includes all replicate extracts and runs of an isolate, Sample:Gene represents random variation between amplifications of different genes in each sample, and Sample:TechRep describes variation in amplification of technical replicates of each sample. Sample:Gene:TechRep is the residual term, relating to random variation in the amplification of each gene in each technical replicate of each sample.

* is the crossing operator (A*B = A+B+A:B), / is the nesting operator (A/B = A+ A:B), and : denotes an interaction term.

The model was defined by the VCOMPONENTS directive of Genstat and fitted by residual maximum likelihood (REML) using the REML directive with optimisation by average information.

Differences between the *Cyp51* and *Tub2* genes were estimated for each combination of Gene.Lineage (variation in log_e_ gene number between lineages) and Gene.Clone.Isolate (variation in log_e_ gene number between isolates within the clonal lineages from JIC’s glasshouses). These differences were back-transformed by the exponential function to obtain an estimate and confidence interval of the number of *Cyp51* genes in each glasshouse clone and each of the other, diverse isolates.

**References**

Bio-Rad Laboratories, I. Droplet Digital PCR: Applications Guide.

Sherwood, J. E., and Somerville, S. C. 1990. Sequence of the *Erysiphe graminis* f. sp. *hordei* gene encoding beta-tubulin. Nucleic Acids Res. 18:1052-1052.
