# Supplementary material for: Multiple routes to fungicide resistance: Interaction of Cyp51 gene sequences, copy number and expression
Source: Mol Plant Pathol. 2024 Sep 20;25(9):e13498. doi: 10.1111/mpp.13498 (PMC11415427; doi:10.1111/mpp.13498)
Supplement: Supplementary file 6 — Table S4. Statistical analysis of data on Cyp51 gene expression in Blumeria graminis f. sp. tritici. [file MPP-25-e13498-s008.docx]

**Table S4.** Statistical analysis of data on *Cyp51* gene expression in *Bgt* obtained by quantitative reverse transcriptase PCR (qRT-PCR).

| a) Variance components of random effects ^a^ | | |
| --- | --- | --- |
| **Random term** | **Component** | **s.e.** |
| Experiment:Gene | 0.4064 | 0.1255 |
| Isolate:Gene | 0.0570 | 0.0482 |
| Experiment:Isolate:Gene | 0.5075 | 0.0583 |
| Isolate:BiolRep | 1.1576 | 0.1594 |
| Isolate:BiolRep:Gene | 0.0222 | 0.0216 |
| Residual | 0.550 | 0.0229 |

| b) Analysis of variance of fixed effects | | | | |
| --- | --- | --- | --- | --- |
| **Fixed term** | **F** | **n.d.f.** | **d.d.f.** | **P(F)** |
| Lineage | 2.87 | 59 | 175.4 | <0.001 |
| GeneGroup | 2.54 | 1 | 29.0 | 0.1 |
| Gene | 11.80 | 2 | 29.5 | <0.001 |
| Lineage:GeneGroup | 2.34 | 59 | 144.8 | <0.001 |

^a^ Abbreviations: s.e., standard error; n.d.f., numerator degrees of freedom; d.d.f., denominator degrees of freedom, P(F); F-test probability.

**Notes**

The variable analysed was *C_q_*.log(*E*), using the *C_q_* value and primer efficiency (*E*) for each PCR amplification reaction. Natural logarithms were used here. If the threshold concentration of DNA detected by the qRT-PCR machine is *k* and the initial concentration of cDNA is *D*, then as a result of successive amplification steps with primer efficiency *E*,

$$k=D.E^{C_{q}}\Rightarrow\log k=C_{q}\log E+\log D$$

As *k* is a constant, log *D* is proportional to *C_q_* log *E*, with lower values of *C_q_* log *E* implying higher values of initial DNA concentration *D*. Nine data points with primer efficiency lower than 1.6 and one with PE greater than 2.2 were excluded, as were two data points for β-tubulin, one of the control genes, which had unusually low *C_q_* values and thus very high residuals.

The mean expression of the three reference genes in each isolate was very highly correlated, with Pearson correlation coefficients of 0.99 between GAPDH and each of the other two genes, and 0.98 between actin and tubulin. Hence the three reference genes can be grouped together in a single factor.

The variable *C_q_* log *E* was used in fitting the following linear mixed model:

Fixed effects: Lineage + GeneGroup / Gene + Lineage : GeneGroup

Random effects: Experiment : Gene + (Isolate : Gene) / Experiment + (Isolate : BiolRep) / Gene

Gene refers to the four genes studied, *Cyp51* and the three reference genes encoding actin, β-tubulin and GAPDH (Glyceraldehyde 3-phosphate dehydrogenase). GeneGroup contrasts the expression of *Cyp51* to the mean expression of the three reference genes. The Lineage and Isolate terms are defined in Table S2. BiolRep relates to biological replicates in which mRNA was extracted from more than one culture of an isolate. The Experiment term relates to the series of experiments done by various authors of this paper in the USA and the UK. See Table S3 for the notation used in describing the model.

The term which describes variation between *Bgt* strains in gene expression is Lineage:GeneGroup. Differences between *C_q_* log *E* for *Cyp51* and its mean for the reference genes were estimated for each combination of Lineage:GeneGroup and back-transformed by the exponential function to estimate the expression of *Cyp51* in proportion to the mean expression of the reference genes in each of the UK glasshouse clones and the other, diverse isolates.

The term that describes variation between isolates within the JIC glasshouse clonal lineages is Lineage:Isolate:GeneGroup. It was not possible to include this term among the fixed effects because the model exceeded the computer’s memory capacity. When this term was treated as a random effect, it had a small negative variance component of −0.0395 ± 0.0776, indicating that it did not contribute a significant amount of variation. It was therefore omitted from the model altogether.
