# Supplementary material for: Multiple routes to fungicide resistance: Interaction of Cyp51 gene sequences, copy number and expression
Source: Mol Plant Pathol. 2024 Sep 20;25(9):e13498. doi: 10.1111/mpp.13498 (PMC11415427; doi:10.1111/mpp.13498)
Supplement: Supplementary file 7 — Table S5. Traits of Blumeria graminis f. sp. tritici isolates. [file MPP-25-e13498-s010.docx]

**Table S5.** Traits of *Blumeria graminis* f.sp. *tritici* isolates characterized for fungicide sensitivity in relation to *Cyp51* allele.

| **Source**  **& CYP51 allele(s)** | **CYP51 genotype** | **Number of isolates** | | **Mean & *range* of tebuconazole ED50** | | **Mean & *range* of prothioconazole ED50** | | **Mean number & *range* of *Cyp51* genes** | **Mean & *range* of *Cyp51* expression cf reference genes (qRT-PCR)** | **Mean & *range* of *Cyp51* transcription (10^3^ FPKM)** |
| --- | --- | --- | --- | --- | --- | --- | --- | --- | --- | --- |
|  |  | Field | Glasshouse^a^ | US tests (mg L^−1^) | UK tests  (mL ha^−1^)^b^ | US tests  (mg L^−1^) | UK tests  (mL ha^−1^)^b^ |  |  |  |
| **USA^c^** |  |  |  |  |  |  |  |  |  |  |
| Y136 S509 | Y+S | 227 |  | 0.70 | 11 | 31 | 47 | 1.8 | 0.19 | 6.5 |
|  |  |  |  | *0.12−7.4* | *9.6−12* | *6.8−320* | *37−64* | *0.6−4.0* | *0.059–0.48* | *4.4−10* |
| F136 S509 | F+S | 107 |  | 3.6 | 42 | 64 | 150 | 1.7 | 0.27 | 25 |
|  |  |  |  | *0.36−16* |  | *7.8−210* |  | *0.7−3.0* | *0.16–0.47* | *21−29* |
| Y/F136 S509 | Het+S | 33 |  | 3.3 |  | 82 |  | 2.6 | 0.38 |  |
|  |  |  |  | *1.4−10* |  | *41−240* |  | *2.4−2.9* | *0.32–0.43* |  |
| **UK**^d^ |  |  |  |  |  |  |  |  |  |  |
| F136 S509 | F+S | 8 | 22 | 31 | 78 | 586 | 200 | 3.4 | 0.85 | 130 |
|  |  |  |  | *14−51* | *56−140* | *380−850* | *110−440* | *2.5−3.9* | *0.58–2.3* | *95−220* |
| F136 T509 | F+T |  | 1 |  | 230 |  | 200 | 2.3 | 0.78 | 51 |
| Y/F136 S/T509 | Het+Het | 8 | 7 | 68 | 170 | 610 | 300 | 3.3 | 0.49 | 52 |
|  |  |  |  | *38−120* | *66−340* | *310−920* | *160−570* | *2.2−5.3* | *0.28–1.1* | *19−94* |
| **Reference** |  |  |  |  |  |  |  |  |  |  |
| JIW11 (UK 1985) | Y+S |  |  | 7.1 | 33 | 500 | 71 | 2.1 | 0.22 | 24 |
| 94202 (CH 1994) | Y+S |  |  |  | 30 |  | 120 | 1.9 | 0.47 | 28 |
| Fel09 (D 1998) | F+S |  |  | 14 | 36 | 460 | 120 | 2.8 | 0.53 | 88 |
| 96224 (CH 1996) | Het+Het |  |  |  | 29 |  | 130 | 1.6 | 0.15 | 23 |

^a^ Each of the four clones collected in JIC’s glasshouses is counted here as one isolate.

^b^ In UK tests, tebuconazole formulated as Folicur and prothioconazole as Proline 275 (both Bayer Crop Science, Leverkusen, Germany).

^c^ Briefly, susceptible wheat seedlings (cultivar ‘Jagalene’) were sprayed to runoff with a particular fungicide concentration. The next day, leaves were segmented, placed atop water agar amended with benzimidazole (50 mg L^-1^), and inoculated with a single *Bgt* isolate. This assay yielded four leaf segment ratings (0, 1, 2, or 3, with 0 being no disease and 3 being maximal disease) per concentration per fungicide for each isolate evaluated. After initial tests, fungicide concentrations were increased to capture the higher resistance of the UK isolates. Tebuconazole concentrations of 0, 20, 40, 60, 80, 100, 120, 140, 160, 180, 200, and 220 mg L^-1^ and prothioconazole concentrations of 0, 50, 100, 200, 300, 400, 500, 600, 700, 800, 900, and 1000 mg L^-1^ were used. Isolates JIW11 and Fel09 were evaluated using the original concentrations presented in Meyers et al. (2019).

Metconazole sensitivity was evaluated in the USDA lab using a smaller sample of isolates from both countries, and ED50s of metconazole were correlated with those of tebuconazole and prothioconazole (E. Meyers’ thesis).

^d^ Trays of seedlings of the wheat cultivar ‘Cerco’ were sprayed four times to obtain the target dose, and detached leaves were placed on water-agar containing 50 mg L^−1^ benzimidazole. Proline 275 (active ingredient 275 g L^−1^ prothioconazole; Bayer Crop Science UK) was sprayed at 2-fold intervals of 0, 22.5, 45, 90, 180, 360 and 720 mL ha^−1^ and Folicur (tebuconazole 250 g L^−1^; Bayer Crop Science UK) at 1.9-fold intervals of 0, 16, 30, 58, 109, 208, 395 and 750 mL ha^−1^. In each test, a set of 2.5-cm-long leaf segments comprising three replicates of each dose were inoculated in a settling tower and mildew colonies that were beginning to sporulate were counted eight days after inoculation. Estimation of median effective doses (ED50) for each isolate is described in Table S6. ED50 estimates were used to compare isolates by geography, *Cyp51* sequence, *Cyp51* copy number, *Cyp51* expression and sporulation by linear mixed modelling using the VCOMPONENTS and REML directives of Genstat. Linear modelling was also used to evaluate the correlations between levels of sensitivity to the different DMIs for each isolate collection.

**Reference**

Meyers, E., Arellano, C., and Cowger, C. 2019. Sensitivity of the U.S. *Blumeria graminis* f. sp. *tritici* population to demethylation inhibitor fungicides. Plant Dis. 103:3108-3116.
