# Supplementary material for: Multiple routes to fungicide resistance: Interaction of Cyp51 gene sequences, copy number and expression
Source: Mol Plant Pathol. 2024 Sep 20;25(9):e13498. doi: 10.1111/mpp.13498 (PMC11415427; doi:10.1111/mpp.13498)
Supplement: Supplementary file 8 — Table S6. Statistical analysis of median effective doses (ED50) of tebuconazole and prothioconazole. [file MPP-25-e13498-s011.docx]

**Table S6.** Statistical analysis of variation in median effective doses (ED50) of tebuconazole and prothioconazole between groups of *Bgt* isolates defined by CYP51 sequence and country of origin.

| a) Variance components of random effects ^a^ | | |
| --- | --- | --- |
| **Random term** | **Component** | **s.e.** |
| Lineage | 0.07612 | 0.02089 |
| Lineage:Lab | 0.00611 | 0.00985 |
| Lineage:Fungicide | 0.00931 | 0.01153 |
| Lineage:Lab:Fungicide | 0.03630 | 0.01168 |

| b) Analysis of variance of fixed effects | | | | |
| --- | --- | --- | --- | --- |
| **Fixed term** | **F** | **n.d.f.** | **d.d.f.** | **P(F)** |
| Lab | 113.59 | 1 | 21.3 | <0.001 |
| Fungicide | 1012.54 | 1 | 48.7 | <0.001 |
| Group | 25.35 | 8 | 59.1 | <0.001 |
| Lab:Fungicide | 281.29 | 1 | 30.3 | <0.001 |
| Lab:Group | 19.25 | 5 | 23.6 | <0.001 |
| Fungicide:Group | 5.36 | 8 | 54.1 | <0.001 |
| Lab:Fungicide:Group | 1.24 | 5 | 32.6 | 0.3 |

c) Mean log10 ED50 (± standard error) of tebuconazole and prothioconazole for groups of isolates

| **Group** | **log10 ED50** | |
| --- | --- | --- |
|  | **Tebuconazole** | **Prothioconazole** |
| USA Y+S | 0.530 ± 0.076 | 1.715 ± 0.076 |
| USA F+S | 1.102 ± 0.155 | 1.939 ± 0.149 |
| USA Het +S | 0.519 ± 0.146 | 1.915 ± 0.146 |
| UK F+S | 1.698 ± 0.094 | 2.546 ± 0.094 |
| UK F+T | 2.367 ± 0.357 | 2.311 ± 0.357 |
| UK Het+Het | 2.016 ± 0.096 | 2.629 ± 0.096 |
| Fel09 & JIW11 (Y+S) | 1.236 ± 0.244 | 2.310 ± 0.244 |
| Fel 09 (F+S) | 1.350 ± 0.324 | 2.366 ± 0.324 |
| 96224 (Het+Het) | 1.464 ± 0.357 | 2.109 ± 0.357 |

d) Significance tests of differences between log10 ED50 of groups of *Bgt* isolates

| **Comparison** | **d.f.** | **Tebuconazole** | | | |  | **Prothioconazole** | | | |
| --- | --- | --- | --- | --- | --- | --- | --- | --- | --- | --- |
|  |  | **Differ-ence** | **RF** ^b^ | **t** | **P(t)** |  | **Differ-ence** | **RF** | **t** | **P(t)** |
| US, F+S – Y+S ^c^ | 48 | 0.572 | 3.7 | 3.33 | 0.002 |  | 0.224 | 1.7 | 1.34 | 0.2 |
| US, Het+S – Y+S ^d^ | 51 | 0.674 | 4.7 | 3.66 | <0.001 |  | 0.418 | 2.6 | 2.27 | 0.03 |
| US, Het+S – F+S ^d^ | 51 | 0.034 | 1.1 | 0.17 | 0.9 |  | 0.103 | 1.3 | 0.49 | 0.6 |
| UK, F+T – F+S ^e^ | 29 | 0.475 | 3.0 | 2.81 | 0.009 |  | 0.004 | 1.0 | 0.02 | 1.0 |
| UK, Het+Het – F+S ^c^ | 48 | 0.318 | 2.1 | 2.37 | 0.02 |  | 0.082 | 1.2 | 0.61 | 0.5 |
| UK, Het+Het – F+T ^e^ | 29 | 0.140 | 1.4 | 0.83 | 0.4 |  | 0.163 | 1.5 | 0.96 | 0.3 |
| UK F+S – US F+S ^c^ | 48 | 0.595 | 3.9 | 3.28 | 0.002 |  | 0.608 | 4.1 | 3.45 | 0.001 |

^a^ Abbreviations: s.e., standard error; d.f., degrees of freedom; n.d.f., numerator degrees of freedom; d.d.f., denominator degrees of freedom; P(F), F-test probability; P(t), t-test probability.

^b^ RF: resistance factor, i.e. the proportion by which the ED50 of the first group in the Comparison column exceeds that of the second group.

^c‑e^ t-tests done on data from ^c^ both labs, ^d^ USDA data or ^e^ JIC data.

**Notes**

***Analysis of variance between labs, fungicides and groups of isolates***

The variable analysed was log10 of the ED50 of each fungicide measured by different methods in the two labs. Data were fitted to the following linear model:

Fixed effects: Lab * Fungicide * Group

Random effects: Lineage / (Lab * Fungicide)

Lineage is defined in Tables S2 and S3. JIC glasshouse clones 1, 2, 3 and 5 were each treated as a single lineage.

Groups include the four reference isolates with 94202 and JIW11 in one group with 96224 and Fel09 each in a group of their own.

***Estimation of ED50s in UK tests***

In the UK fungicide tests, ED50 were calculated for each isolate in each batch of tests with each fungicide by nonlinear regression of colony numbers (*N*) on each leaf against the log-transformed dose (*L*) using a logistic model:

1. $N_{ibfr}=U_{ibf}\left[ 1+\exp\left\{ -S_{f}\left( L_{cf}-M_{ibf} \right) \right\} \right]^{-1}$

where subscript *r* indicates the three replicate leaf segments, *M_ibf_* is log(ED50) of isolate *i* in batch *b* of tests with fungicide *f, U_ibf_* is the mean colony number on untreated leaves without fungicide, *S_f_* is a slope parameter for fungicide *f* and *L_cf_* is the log-transformed concentration *c* of fungicide *f*. The slope parameter *S_f_* is difficult to estimate for a genetically uniform organism because it reflects random variation in the amount of fungicide on each leaf, not genetic variation in the responses of the organism itself (Brown 1991) . In a preliminary analysis of responses to each fungicide, the model above was used to estimate *S_f_* separately for each isolate and batch, then the median value of *S_f_* was used as a fixed parameter in refitting the model to estimate the intercept *U_ibf_*, the log(ED50) *M_ibf_* and the standard error (SE) of log(ED50) for all tests with that fungicide. Curve-fitting was done with the FITCURVE directive of Genstat (VSN International, Hemel Hempstead, UK).

**Reference**

Brown, J. K. M. 1991. Statistical analysis of the response of powdery mildews to fungicides. Pages 161-175 in: Integrated Control of Cereal Mildews: Virulence Patterns and Their Change. Eds: Jørgensen, J. Risø National Laboratory, Roskilde, Denmark.
