# Supplementary material for: Multiple routes to fungicide resistance: Interaction of Cyp51 gene sequences, copy number and expression
Source: Mol Plant Pathol. 2024 Sep 20;25(9):e13498. doi: 10.1111/mpp.13498 (PMC11415427; doi:10.1111/mpp.13498)
Supplement: Supplementary file 9 — Table S7. US Blumeria graminis f. sp. tritici isolates genotyped from each of 27 fields. [file MPP-25-e13498-s013.docx]

**Table S7**. Number of U.S. *Blumeria graminis* f. sp. *tritici* isolates genotyped from each of 27 fields where mildewed wheat samples were collected in 2013 and 2014.

| **State** | **Field number**^a^ | **Number of isolates** |
| --- | --- | --- |
| Nebraska (NE) | 1 | 11 |
| Kansas (KS) | 2 | 18 |
| Oklahoma (OK) | 3 | 6 |
|  | 4 | 9 |
|  | 5 | 9 |
| Missouri (MO) | 6 | 15 |
| Arkansas (AR) | 7 | 12 |
| Mississippi (MS) | 8 | 19 |
| Alabama (AL) | 9 | 16 |
| Florida (FL) | 10 | 17 |
| Georgia (GA) | 11 | 23 |
|  | 12 | 14 |
|  | 13 | 14 |
| Michigan (MI) | 14 | 10 |
| Ohio (OH) | 15 | 18 |
|  | 16 | 14 |
| New York (NY) | 17 | 9 |
|  | 18 | 5 |
|  | 19 | 16 |
|  | 20 | 12 |
| Pennsylvania (PA) | 21 | 15 |
|  | 22 | 15 |
| Virginia (VA) | 23 | 12 |
| North Carolina (NC) | 24 | 12 |
|  | 25 | 15 |
|  | 26 | 15 |
|  | 27 | 12 |
| TOTAL |  | 363 |

^a^ Field numbers correspond to those in Meyers et al. (2019), where there is a table of locations (nearest towns). See the present Fig. 2 for approximate field locations. The states were grouped into regions distinguished by microsatellite marker frequencies (Cowger et al. 2016) or sensitivity to DMIs (Meyers et al. 2019). Isolates were maintained on plates of detached wheat leaf segments (susceptible cvs. ‘Chancellor’ or ‘Jagalene’) on 0.5% agar amended with benzimidazole at 50 mg L^-1^, and transferred to fresh leaf segments every 8-11 days as previously described (Meyers et al. 2019).

From the 363 isolates, a subset of 30 was selected for *Cyp51* expression and copy number assays. The 30 isolates represented all 15 states from which the larger isolate collection had originated, and all available combinations of three factors: variant at codon 136, and high or low sensitivity to each of the two fungicides. Each genotype-phenotype combination observed in a US state was included in the 30-isolate subset, and all available isolates with heteroallelic *Cyp51* were also included.

Reference

Meyers, E., Arellano, C., and Cowger, C. 2019. Sensitivity of the U.S. *Blumeria graminis* f. sp. *tritici* population to demethylation inhibitor fungicides. Plant Dis. 103:3108-3116.
