# Supplementary material for: Multiple routes to fungicide resistance: Interaction of Cyp51 gene sequences, copy number and expression
Source: Mol Plant Pathol. 2024 Sep 20;25(9):e13498. doi: 10.1111/mpp.13498 (PMC11415427; doi:10.1111/mpp.13498)
Supplement: Supplementary file 10 — Table S8. Primers for amplification and sequencing of Cyp51 in the United States. [file MPP-25-e13498-s014.docx]

**Table S8.** Overlapping primers used in the US to sequence the entirety of the *Cyp51* gene in 363 *Blumeria graminis* f. sp. *tritici* isolates using an amplicon-sequencing approach.

Two pools of primers were used to sequence the gene in overlapping fragments of 173 to 275bp. Preparation and sequencing of AmpSeq libraries was completed under the oversight of L. Cadle-Davidson at Cornell University. Sequencing reads were aligned to reference sequences of the amplicons as found in the 2013 genome assembly of the Bgt reference isolate 96224 (GenBank accession GCA_000418435.1), using BWA v07.5a-r405 (Li 2011). Variants were called using freebayes v1.0.2-15-g357f175 (Garrison and Marth 2012).

| **Primer Name** | **Primer Sequence (5’ to 3’)** | **Forward Primer Position** |
| --- | --- | --- |
| 51-01b_F | CATACTTCATGGCGAGCGG | -597 |
| 51-01b_R | ACACATGTAATCCTCCATAACAGC | -597 |
| B51-02F | TTTTCCAACCTAGAGGCACTG | -486 |
| B51-02R | CGAAACTGATTTCCCGTTTT | -486 |
| A51-03F | CGCTCATAGCACGAATTTCA | -313 |
| A51-03R | GTAATATGTTTCCAGCGGTCTGTG | -313 |
| 51-03b_R | GTTTCCAGCGGTCTGTGATT | -313 |
| B51-04F | GGATTGAACTCCGCCGACTA | -117 |
| B51-04R | AGTAGCTGCTTCAATACATTCAAGA | -117 |
| A51-05F | CGTTGGCTAGTGGAATTATAAGTTTAT | 68 |
| 51-05b_R | TCCCCAGAATATATCAATGGCA | 68 |
| B51-06F | AATCCCAAGCCAAGGTCAGT | 233 |
| B51-06R | ACATCAGTCCCGAAGACAGG | 233 |
| 51-07b_F | ACTGAGGGATGTTAATGCTGAAG | 387 |
| 51-07b_R | AAAGCTCTCCACTTCATTTTGG | 387 |
| B51-08F | GCCTTCCGCTCTTATGTACCT | 575 |
| B51-08R | GCATGAAATTGATTGGGGTAA | 575 |
| A51-09F | TTGGCAGTTTTGTATCATGACC | 755 |
| A51-09R | GGGAGTGCCATCTTTGTAGG | 755 |
| B51-10F | GTGGCAATTAATGCGCTCTT | 938 |
| B51-10R | ATTTAACAGGAGGTAATTCTGATCC | 938 |
| 51-11b_F | TGAAGAACTCTACCAAGAACAGC | 1094 |
| 51-11b_R | CTGGAACGGGCATTGGATTC | 1094 |
| 51-12b_F | GTGAAAGAGGTCCTCCGTCT | 1191 |
| 51-12b_R | CTTGCTGCCCCTGTGCTAA | 1191 |
| A51-13F | AGGCACGGATATGGAGGATG | 1400 |
| A51-13R | CCATTGGCCGAGAAAACATACT | 1400 |
| B51-14F | CAACGGTGCAATTAGTTACAATCA | 1519 |
| B51-14R | AGCTTGTAATCTAATCATCTCCAAACT | 1519 |

**References**

Garrison, E., and Marth, G. 2012. Haplotype-based variant detection from short-read sequencing. arXiv:1207.3907 [q-bio.GN].

Li, H. 2011. A statistical framework for SNP calling, mutation discovery, association mapping and population genetical parameter estimation from sequencing data. Bioinformatics 27:2987-93.
