# Supplementary material for: Multiple routes to fungicide resistance: Interaction of Cyp51 gene sequences, copy number and expression
Source: Mol Plant Pathol. 2024 Sep 20;25(9):e13498. doi: 10.1111/mpp.13498 (PMC11415427; doi:10.1111/mpp.13498)
Supplement: Supplementary file 11 — Table S9. Primers for amplification and sequencing of Cyp51 in the UK. [file MPP-25-e13498-s006.docx]

Table S9. Primers used in the UK for amplification and sequencing of the *Bgt Cyp51* gene.

Amplicons 2 and 6 were amplified and sequenced in *Bgt* isolates to identify amino acids 136 and 509. PCR was done in 25-µL reactions containing 1x Q5® reaction buffer, 200 µM dNTPs, 0.5 µM each forward and reverse primers, 0.5 U Q5® High-Fidelity DNA polymerase (New England BioLabs Inc., UK), 3% DMSO and 30 ng DNA, made up to 25 µL with water. Thermocycling conditions were 98 °C for 30 seconds, followed by 35 cycles of 98 °C for 10 seconds, 60 °C for 15 seconds, and 72 °C for 20 seconds, with a final extension at 72 °C for 2 minutes. PCR products were purified using a QIAGEN QIAquick PCR Purification Kit according to the manufacturer’s instructions and quantified using a NanoDrop2000 Spectrophotometer. They were then sent to Eurofins Genomics for DNA sequencing using primer Blumeria_CYP51_Amp2.1.

| Primer name | Primer sequence (5’ to 3’) | Melting temperature (°C) | Amplicon location and size |
| --- | --- | --- | --- |
| Blumeria_CYP51_Amp1.1 | ATGGGAAAACCAGAAAGCT | 59.75 | 1-400; 400 bp |
| Blumeria_CYP51_Amp1.2 | TAACATCCCTCAGTTTTCCA | 59.25 |  |
| Blumeria_CYP51_Amp2.1 | TTTCACTTTCATATTACTGGGTAA | 58.86 | 309-709; 401 bp |
| Blumeria_CYP51_Amp2.2 | TGAAGCGGTATATATCGTAATTTC | 59.32 |  |
| Blumeria_CYP51_Amp3.1 | AAATAAATGCGACGATTTTCG | 58.69 | 625-1014; 390 bp |
| Blumeria_CYP51_Amp3.2 | GCGATCATCATATGTGCAAT | 58.94 |  |
| Blumeria_CYP51_Amp4.1 | TATCATGTGGCAATTAATGCG | 59.61 | 934-1301; 368 bp |
| Blumeria_CYP51_Amp4.2 | GGGAATGGGTCTTAGGTATT | 59.17 |  |
| Blumeria_CYP51_Amp5.1 | GAAAAGTAAAGAATCCAATGCC | 58.72 | 1239-1544; 306 bp |
| Blumeria_CYP51_Amp5.2 | TAATTGTAACTAATTGCACCGTT | 59.59 |  |
| Bg_CYP51Amp6.1R | AAAATTCGATTATGGGTATGGAT | 58.92 | 1423-1696; 274 bp |
| Bg_CYP51Amp6.2R | AGTAAACTTAACACTCCGTTTT | 58.96 |  |
