# Supplementary material for: Multiple routes to fungicide resistance: Interaction of Cyp51 gene sequences, copy number and expression
Source: Mol Plant Pathol. 2024 Sep 20;25(9):e13498. doi: 10.1111/mpp.13498 (PMC11415427; doi:10.1111/mpp.13498)
Supplement: Supplementary file 12 — Table S10. Primers and product sizes for estimating Cyp51 copy number. [file MPP-25-e13498-s001.docx]

Table S10: Primer sequences and PCR product sizes for estimating *Cyp51* copy number using *Tub2* as a reference gene. *Tub2* primers were designed using GenBank ID JQ268163.1, a partial gene sequence.

| Target gene | Primer sequence 5’ to 3’ | Product size | Amplicon location in gene |
| --- | --- | --- | --- |
| *Cyp51* | TTTCATGCTTCACTGGGCAC | 116 bp | 802-917 |
|  | CAGTTTCTTTCTGCGTCCGA |  |  |
| *Tub2* | AGAACATGATGGCAGCCTCC | 88 bp | 927-1014 |
|  | GCATGCGTATAAAACGTGCAG |  |  |

Copy number of *Cyp51* was estimated relative to that of the β-tubulin gene (*Tub2*; GenBank accession JQ268163.1), which is known to be a single copy gene in *B. graminis* (Sherwood and Somerville 1990). PCR was set up in 22 µL reactions containing 1x QX200^TM^ ddPCR^TM^ EvaGreen Supermix (BIO-RAD UK), 200 nM forward primer, 200 nM reverse primer, 5 U NcoI-HF®, and 3 ng DNA. Restriction enzyme NcoI-HF® (New England BioLabs Inc., UK) was added to the PCR mix for digestion before PCR commenced to separate any tandem copies. Thermocycling utilized the BIO-RAD QX200^TM^ ddPCR^TM^ EvaGreen Supermix protocol: 95 °C for 5 minutes, followed by 40 cycles of 95 °C denaturation for 30 seconds, and 60 °C annealing and extension for 1 minute. This was followed by a signal stabilisation step at 4 °C for 5 minutes then 90 °C for 4 minutes. After thermocycling, the 96-well plate was placed in a BIO-RAD QX200^TM^ Droplet Reader for absolute quantification of each reaction using BIO-RAD QuantaSoft^TM^ software. A medium threshold was applied to each reaction to delineate a boundary between positive and negative droplets, containing or lacking at least one amplified fragment respectively. Readings with at least 10,000 total droplets were accepted and any reactions producing fewer droplets were re-run. The number of *Cyp51* genes was estimated for each field isolate and each lineage and isolate of the glasshouse sample. Details of the statistical methods are in Table S3.

**Reference**

Sherwood, J. E., and Somerville, S. C. 1990. Sequence of the *Erysiphe graminis* f. sp. *hordei* gene encoding beta-tubulin. Nucleic Acids Res. 18:1052-1052.
