# Supplementary material for: Multiple routes to fungicide resistance: Interaction of Cyp51 gene sequences, copy number and expression
Source: Mol Plant Pathol. 2024 Sep 20;25(9):e13498. doi: 10.1111/mpp.13498 (PMC11415427; doi:10.1111/mpp.13498)
Supplement: Supplementary file 13 — Table S11. Primers and other details for reverse transcription‐quantitative PCR estimation of Cyp51 expression. [file MPP-25-e13498-s012.docx]

**qRT-PCR**

Table S11: *Cyp51* expression estimation: qRT-PCR primer sequences, PCR product sizes, annealing temperatures, and primer efficiencies, and RNA-seq protocol

| Target gene | Primer name | Primer sequence (5’ to 3’) | Product size | Annealing temperature | Efficiency |
| --- | --- | --- | --- | --- | --- |
| *Tub2* | TUBb_x12a_F | ACATGCTCTGCTATTTTCCG | 105 bp | 58 °C | 1.90 |
|  | TUBb_1a_R | TGGAATCCACTCAACAAAGT |  |  |  |
| *Actin* | ACT_x12a_F | CGAGCTGTTTTCCCATC | 125 bp | 54 °C | 1.89 |
|  | ACT_2a_R | TATCTAAGAGTCAGAATACCA |  |  |  |
| *Cyp51* | cyp51_x12b_F | GAATCCCAAGCCAAGTAC | 122 bp | 60 °C | 1.89 |
|  | cyp51_x_splice_R | GCATTAACATCCCTCAGTT |  |  |  |
| *GAPDH* | Forward | CGTATAGGCCGCATTGTCTT | 117 bp | 60 °C | 1.87 |
|  | Reverse | TCCTCGACTGTTTGGATGTCT |  |  |  |

aaa

In both UK and US labs, to produce RNA, epidermal peels were obtained by painting detached, mildew-infected leaf segments with cellulose acetate, then stripping the epidermis from the leaf (Zhang et al. 2004) and grinding it with a pestle, mortar, sand, and liquid nitrogen. RNA was extracted using a QIAGEN Rneasy Plant Mini Kit according to the manufacturer’s protocol using buffer RLC. To remove DNA, 20 μL RNA extract was added to 3 U TURBO™ Dnase and 1x TURBO^TM^ Dnase buffer, and incubated for 30 minutes at 37 °C. This was repeated once without adding extra buffer. 0.2 volumes Dnase inactivation reagent was added and the reaction was vortexed then incubated at room temperature for 5 minutes, vortexing briefly every minute. Reactions were centrifuged for 1.5 minutes at 10,000 g, then the supernatant was transferred to a clean Eppendorf tube. RNA was quantified using a NanoDrop2000 spectrophotometer.

For cDNA synthesis, a QIAGEN QuantiTect Reverse Transcription Kit was used according to the manufacturer’s protocol. Assuming the cDNA synthesis was 100% efficient, cDNA was diluted to 25 ng/µL in the UK and 2 ng/µL in the USA based on RNA NanoDrop readings.

To estimate the level of *Cyp51* gene expression in relation to biological standards, qRT-PCR was performed using primers designed to amplify *Cyp51* and three reference genes expected to have steady rates of expression, encoding β-tubulin (*Tub2* gene), glyceraldehyde-3-phosphate dehydrogenase (GAPDH), and actin. Each isolate was evaluated using three separate extracts as biological replicates and three technical replicates (two in the USA) of each biological replicate per gene. Primers were designed so that the forward primers were located over exon splice sites to make them cDNA-specific. Primer sequences, PCR product sizes, primer annealing temperatures, and primer efficiencies are shown in Table S10, as well as details on the PCR protocols and efficiency calculation methods.

In the USA, to calculate the efficiency of *Tub2* primers, a 10-fold dilution series of one cDNA sample from 46.8 ng µL^–1^ to 0.0468 ng µL^–1^ was used. To calculate primer efficiencies of actin, GAPDH and *Cyp51* primers, a 5-fold dilution series of a second cDNA sample (from 6.88 ng/µL to 0.055 ng/µL) was used because of the amount of cDNA available. qRT-PCR primer efficiency assays were performed in 10 µL reaction volumes including 2 µL diluted cDNA, 1x SsoAdvanced^TM^ universal SYBR® Green supermix, 0.3 µM forward primer, 0.3 µM reverse primer, and made up to 10 µL with water. Thermocycling was performed in a BIO-RAD CFX96 Real-Time System C1000 Thermal Cycler using the following programme: 95 °C for 30 seconds, 40 cycles of 95 °C denaturation for 5 seconds then the appropriate annealing temperature for 30 seconds with a plate read at the end of every cycle. After cycling, a melt curve was produced by increasing the temperature from 65 °C to 95 °C in 0.5 °C increments with 5 seconds allowed per increment. A plate read was taken at every increment. Standard curves and efficiencies were calculated using the BIO-RAD CFX Manager 3.1 software.

After determining that primer efficiencies were within the acceptable range (90-105%), qRT-PCR was performed with cDNA from the isolates listed in Table S1. PCR was performed with the same reaction component quantities as when determining primer efficiencies, except that 4 ng cDNA was used per reaction instead of a dilution series. Two technical replicates per biological replicate were assayed. Quantitation cycle (Cq) values were calculated automatically using the BIO-RAD CFX Manager 3.1 software.

In the UK, qRT-PCR was performed using 10 µL reaction volumes including 50ng cDNA, 1x Luna Universal qPCR mastermix from New England Biolabs, 0.25 µM forward primer, 0.25 µM reverse primer, and made up to 10 µL with water. Thermocycling was performed in a LightCycler480 (Roche) using the following programme: 95 °C for 5 minutes, 45 cycles of 95 °C denaturation for 10 seconds then the appropriate annealing temperature for 30 seconds with a plate read at the end of every cycle. After cycling, a melt curve was produced by Denaturation at 95 °C for 5s followed by cooling to 65°C for 1 minute, the temperature was then increased from 65°C to 97°C in 0.11°C/s increments. A plate read was taken at every increment. Standard curves were analysed using the LightCycler480 software. Raw data output from the LightCycler was converted into the input format of LinRegPCR ususing LC480 conversion application version 2014.1 December 2014 (<https://www.medischebiologie.nl>). Data was analysed using the LinRegPCR version 2021.2 (<https://www.medischebiologie.nl>). The program determined a baseline fluorescence and performed a baseline subtraction. Then a Window-of-Linearity was set and PCR efficiencies per sample were calculated. With the mean PCR efficiency per amplicon, the Cq value per sample and the fluorescence threshold were set to calculate the Cq value. Primer efficiencies (PE) were inspected for each technical replicate of each gene amplification for each isolate, and 10 readings were excluded from the analysis because PE was less than 1.6 or greater than 2.2.

For both labs, the statistical method that employed Cq and PE values to estimate *Cyp51* expression is described in Table S4.

**RNA-seq**

Epidermal peels were collected from leaves infected with the isolates, and RNA was extracted from peels as described above and quantified using an Agilent 2100 Bioanalyser. RNA from each isolate (~3 µg) was sent to GENEWIZ UK (Takeley, UK) for library preparation and sequencing on the Illumina HiSeq platform (2 x 150 bp). Trimmomatic version 0.33 was used to trim adapter sequences and low-quality bases from the ends of the raw reads (Jupe et al. 2013). HISAT2 version 2.1.0 was used to map the transcript reads to the reference genome of *Bgt* isolate 96224 (Wicker et al. 2013). SAMtools version 1.5 was used to pair reads together, then used in conjunction with StringTie version 1.3.4 to assemble the mapped reads into transcripts and estimate transcript abundances, i.e. expression levels, as FPKM (fragments per kilobase of transcript per million mapped reads) values (Danecek et al. 2021). In-house custom Python scripts were then used to extract the *Cyp51* FPKM values (fragments per kb transcript per million mapped reads).

**References**

Danecek, P., Bonfield, J. K., Liddle, J., Marshall, J., Ohan, V., Pollard, M. O., Whitwham, A., Keane, T., McCarthy, S. A., Davies, R. M., and Li, H. 2021. Twelve years of SAMtools and BCFtools. GigaScience 10.

Jupe, F., Witek, K., Verweij, W., Sliwka, J., Pritchard, L., Etherington, G. J., Maclean, D., Cock, P. J., Leggett, R. M., Bryan, G. J., Cardle, L., Hein, I., and Jones, J. D. 2013. Resistance gene enrichment sequencing (RenSeq) enables reannotation of the NB-LRR gene family from sequenced plant genomes and rapid mapping of resistance loci in segregating populations. Plant Journal 76:530-44.

Wicker, T., Oberhaensli, S., Parlange, F., Buchmann, J. P., Shatalina, M., Roffler, S., Ben-David, R., Doležel, J., Šimková, H., Schulze-Lefert, P., Spanu, P. D., Bruggmann, R., Amselem, J., Quesneville, H., Ver Loren van Themaat, E., Paape, T., Shimizu, K. K., and Keller, B. 2013. The wheat powdery mildew genome shows the unique evolution of an obligate biotroph. Nat. Genet. 45:1092-1096.

Zhang, Z., Henderson, C., and Gurr, S. J. 2004. *Blumeria graminis* secretes an extracellular catalase during infection of barley: potential role in suppression of host defence. Mol. Plant Pathol. 5:537-547.
