# Supplementary material for: Multiple routes to fungicide resistance: Interaction of Cyp51 gene sequences, copy number and expression
Source: Mol Plant Pathol. 2024 Sep 20;25(9):e13498. doi: 10.1111/mpp.13498 (PMC11415427; doi:10.1111/mpp.13498)
Supplement: Supplementary file 14 — Table S12. Statistical analysis of US isolate sporulation. [file MPP-25-e13498-s004.docx]

**Table S12.** Experimental protocol and statistical analysis of sporulation of US *Bgt* isolates in relation to CYP51 sequence.

To assess a possible relationship between *Cyp51* genotype and fitness, sporulation as a component of fitness was evaluated for 351 US isolates (Meyers et al. 2019). Five replicate leaf segments with approximately 20 colonies each were obtained for each isolate. To avoid clumping due to hydrophobicity, colonies were collected into tubes of 100% ethanol at 8 days post-inoculation. The ethanol was then evaporated from the samples using a SpeedVac. Dry, pelleted conidia were resuspended in 100 μL of pure, white, light mineral oil by vortexing. Conidia were counted in the 25 innermost squares of a hemocytometer.

Mean spore production per leaf segment was calculated for each variant using a mixed model (below) with genotype as a fixed effect and isolate within genotype as a random effect; a log10 transformation of spore counts per haemocytometer was applied to minimise heteroscedasticity.

The mean sporulation per leaf of F+S isolates was 19% lower than in Het+S isolates and 16% lower than in Y+S (Fig. S1). There was very wide variation between isolates of each genotype and between replicate leaves of each isolate, so even these large differences were not statistically significant (*P* = 0.2).

| a) Variance components of random effects | | |
| --- | --- | --- |
| **Random term** | **Component** | **s.e.** |
| Isolate | 0.1145 | 0.0102 |
| Leaf (residual) | 0.102 | 0.0037 |

| b) Analysis of variance of fixed effects | | | | |
| --- | --- | --- | --- | --- |
| **Fixed term** | **F** | **n.d.f.** | **d.d.f.** | **P(F)** |
| Genotype | 1.49 | 2 | 349.3 | 0.2 |

The variable analysed was log10 of spores per colony. Spores were counted in 25 squares of a haemocytometer then multiplied by 10^5^ to obtain spores per leaf. The number of colonies on each leaf was also counted. A log10 transformation was applied to avoid the usual problem of heteroscedastic residuals when data span several orders of magnitude. The variable log_10_ (spores / colony) was therefore calculated as:

log10 (spores in 25 squares) – log10 (colonies) +5

Values of log10 (spores / colony) were used to fit a model with a fixed effect of CYP51 Genotype and a random effect of Isolate within Genotype. Variation between replicate leaves of the same Isolate was the residual term.

**Reference**

Meyers, E., Arellano, C., and Cowger, C. 2019. Sensitivity of the U.S. *Blumeria graminis* f. sp. *tritici* population to demethylation inhibitor fungicides. Plant Dis. 103:3108-3116.
